# Efficient MoWO_3_/VO_2_/MoS_2_/Si UV Schottky photodetectors; MoS_2_ optimization and monoclinic VO_2_ surface modifications

**DOI:** 10.1038/s41598-020-72990-9

**Published:** 2020-09-28

**Authors:** Mohamed A. Basyooni, Shrouk E. Zaki, Mohamed Shaban, Yasin Ramazan Eker, Mucahit Yilmaz

**Affiliations:** 1 Nanophysics Laboratory, Department of NanoScience and NanoEngineering, Institute of Science and Technology, University of Necmettin Erbakan, Konya, 42060 Turkey; 2 Nanophotonics and Applications Laboratory, Department of Physics, Faculty of Science, Beni-Suef University, Beni Suef, 62514 Egypt; 3 Department of Physics, Faculty of Science, Islamic University in Almadinah Almonawara, Almadinah Almonawara, 42351 Saudi Arabia; 4 Department of Metallurgy and Material Engineering, Faculty of Engineering and Architecture, Necmettin Erbakan University, Konya, 42060 Turkey; 5 Science and Technology Research and Application Center (BITAM), University of Necmettin Erbakan, Konya, 42060 Turkey

**Keywords:** Materials science, Optics and photonics

## Abstract

The distinctive properties of strongly correlated oxides provide a variety of possibilities for modulating the properties of 2D transition metal dichalcogenides semiconductors; which represent a new class of superior optical and optoelectronic interfacing semiconductors. We report a novel approach to scaling-up molybdenum disulfide (MoS_2_) by combining the techniques of chemical and physical vapor deposition (CVD and PVD) and interfacing with a thin layer of monoclinic VO_2_. MoWO_3_/VO_2_/MoS_2_ photodetectors were manufactured at different sputtering times by depositing molybdenum oxide layers using a PVD technique on p-type silicon substrates followed by a sulphurization process in the CVD chamber. The high quality and the excellent structural and absorption properties of MoWO_3_/VO_2_/MoS_2_/Si with MoS_2_ deposited for 60 s enables its use as an efficient UV photodetector. The electronically coupled monoclinic VO_2_ layer on MoS_2_/Si causes a redshift and intensive MoS_2_ Raman peaks. Interestingly, the incorporation of VO_2_ dramatically changes the ratio between A-exciton (ground state exciton) and trion photoluminescence intensities of VO_2_/(30 s)MoS_2_/Si from < 1 to > 1. By increasing the deposition time of MoS_2_ from 60 to 180 s, the relative intensity of the B-exciton/A-exciton increases, whereas the lowest ratio at deposition time of 60 s refers to the high quality and low defect densities of the VO_2_/(60 s)MoS_2_/Si structure. Both the VO_2_/(60 s)MoS_2_/Si trion and A-exciton peaks have higher intensities compared with (60 s) MoS_2_/Si structure. The MoWO_3_/VO_2_/(60 s)MoS_2_/Si photodetector displays the highest photocurrent gain of 1.6, 4.32 × 10^8^ Jones detectivity, and ~ 1.0 × 10^10^ quantum efficiency at 365 nm. Moreover, the surface roughness and grains mapping are studied and a low semiconducting-metallic phase transition is observed at ~ 40 °C.

## Introduction

The current experiments of integrating 2D TMDCs into nano-electronic devices such as MoS_2_, WS_2,_ and black phosphorous still have challenges like low carrier mobility and low photoluminescence (PL) efficiency which limit their further applications in optoelectronics. It is believed that in 2D TMDCs, the Van der Waals (VDWs) interlayer forces are weak and little defects can enhance the intrinsic phonon scattering and lead to better electrical conduction. Charge traps in 2D TMDCs are found to have a direct relationship with the carrier mobility and the output resistances of the electronic devices^1^. Therefore, in order to enhance the Raman intensity, PL intensity, and the charge carrier mobility of the optoelectronic devices, either by an interfacing functional channel material or a functional substrate that modulates the device output in multiple ways, many functional oxides have been used to offer unique properties such as piezoelectricity, strong polarization, and spin injection.

Chemical vapor deposition (CVD) is widely thought to be the most common method for preparing MoS_2_. In which MoO_3_ and sulfur powders are utilized with some inert gases through two or three-zone quartz tubes. The substrate can be maintained at downstream gas flow to grow MoS_2_^2–4^. Nevertheless, this growth technique has several disadvantages through which non-homogenous, tiny flakes, and 2D MoS_2_ in the micro/nanoscale is formed. These disadvantages restrict its application in the industrial optoelectronic devices. On the other hand, methods such as exfoliation approaches, "top-down," have been used to prepare 2D sheets of graphene, MoS_2_, etc. This method including different forms such as micromechanical exfoliation^5^, sonication-assisted liquid-exfoliation^6–8^, shear exfoliation^9,10^ and chemical exfoliation^11,12^. Micromechanical exfoliation still has some disadvantages such as low quality, small-scale production, and high amount of defects^13^. Another drawback is that the exfoliated MoS_2_ must be transferred to a new substratum that handles its scaling and mass production^14^. Nonetheless, for the next generation of optoelectronics and quantum computers, the demand for wafer-scale and homogeneous 2D materials such as MoS_2_ has increased in recent years. Mainly, these methods including atomic layer deposition (ALD)^14,15^, pulsed laser deposition (PLD)^16,17^, thermal evaporation^18,19^, and magnetron sputtering techniques^20–22^. Magnetron sputtering was commonly employed at low cost and with easy control in large-scale for commercial manufacturing.

Strongly correlated oxides are a wide range of materials where the associated electronic, magnetic properties, and spin are strongly correlated with each other. The local spin density approximation (LSDA) has been used to determine the energy band structure of many kinds of materials. However, the LSDA fails to describe the electronic structure of some materials in which the interaction among the electrons is strong (e–e interaction) such as strongly correlated electron systems^23^. Meanwhile, the dominant role of the Coulomb repulsion forces between the electrons in VO_2_ and V_2_O_3_ systems is opening the insulating gap^24,25^. However, when strongly correlated oxides interface with 2D TMDCs, multi-functions such as bandgap, charge transfer, energy transfer, and strain can be tuned^26,27^. Among them, vanadium dioxide (VO_2_); an archetypal strongly correlated functional oxide that exhibits a metal–insulator transition (MIT) above room temperature. At ambient pressure, below the transition temperature (${T}_{c}$), VO_2_ has a monoclinic (M1 phase), with space group ${P2}_{1}/c(\#14)$^28^ and lattice constant of $a\approx 5.75\mathrm{ \AA },\mathrm{ b}\approx 4.53\mathrm{ \AA },\mathrm{ c}\approx 5.38\mathrm{ \AA },\upbeta ={122.6}^{\mathrm{o}}$^29,30^. Above the ${T}_{c}$, VO_2_ adopts a tetragonal rutile (R) structure with space group ${P4}_{2}mnm(\#136)$ and a lattice constants $a=b\approx 4.55\mathrm{ \AA },\mathrm{ c}\approx 2.85\mathrm{ \AA }$^30^.

**Figure 1:**
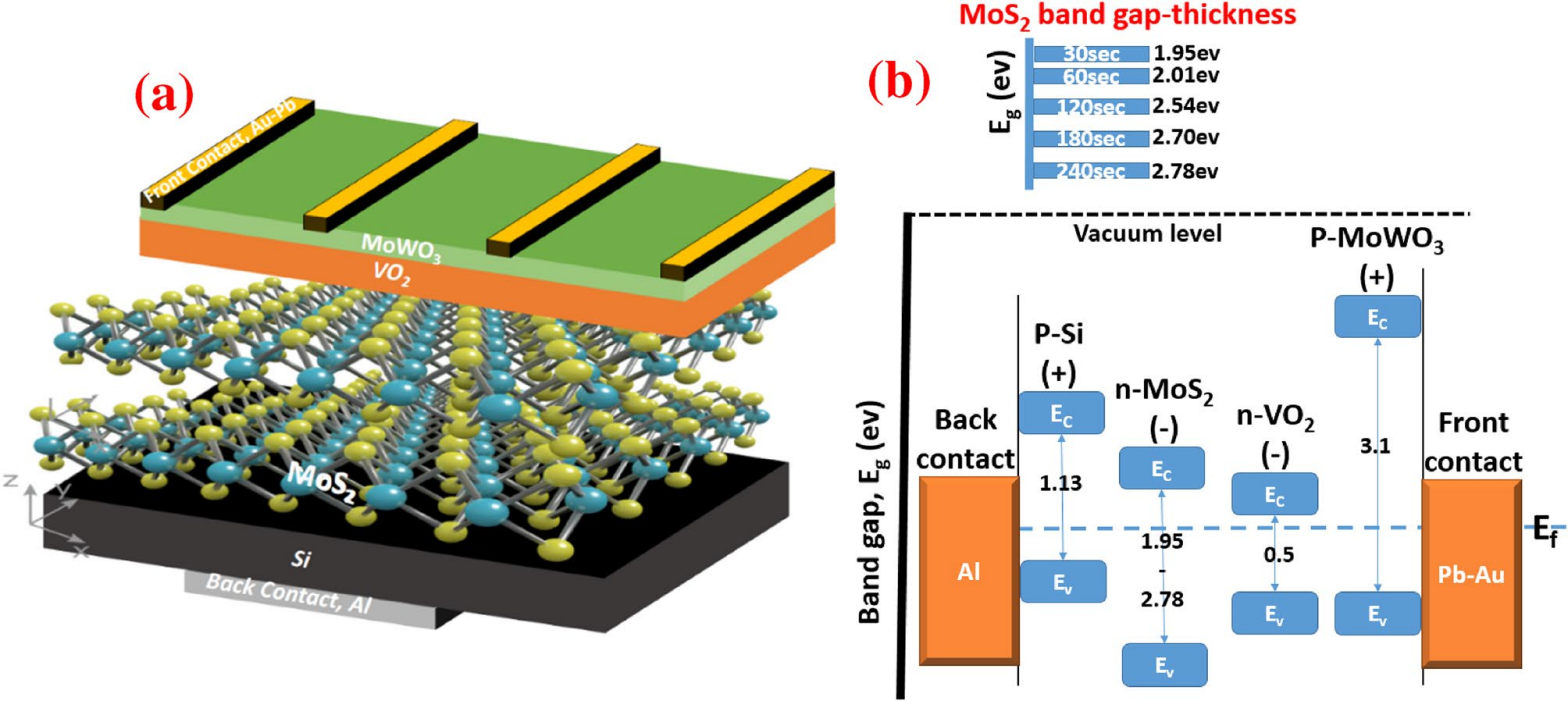
(**a**) schematic diagram of the proposed MoWO_3_/VO_2_/MoS_2_/Si UV photodetector and (**b**) its band alignment with different Mo–O sputtering time (30–240 s).

Due to the superior physical properties at the interface between MoS_2_ and VO_2_ layer, the manufacture of MoS_2_/VO_2_ heterostructure for optoelectronic devices has received considerable attention. However, for optical and optoelectronic products, account should be taken of the manufacturing process of homogenous and scaled-up MoS_2_. Oliva et al. reported the design of Van der Waals MoS_2_/VO_2_ photodetetctor^31^. Nevertheless, in that report, the manufacturing process of MoS_2_ includes several steps related to the micromechanical exfoliation process. The photo-excited carriers transfer in MoS_2_ and VO_2_ was studied, whereas the CVD powder vaporization technique was used for the growth of small flakes of MoS_2_^32^. However, for the easy production of scalable and homogenous MoS_2_-based industrial applications, controllability and optimization of deposition time and growth parameters are highly required. Here, we report the preparation, characterization, and optoelectronic application of MoWO_3_/VO_2_/MoS_2_/Si (p–n–n–p) structure towards 365 nm photodetector at room temperature. Different sputtering times of Mo-O have been controlled followed by sulfurization process and interfacing with a monoclinic VO_2_ layer. Figure 1a shows a schematic diagram of the proposed MoWO_3_/VO_2_/MoS_2_/Si (p–n–n–p) photodetector. Note that most of the previously reported studies were trying to enhance the Raman, PL, optoelectronic of VO_2_–MoS_2_ by controlling the device temperature. However, these attempts did not relay for low temperature-phase transition optoelectronic devices. Here, this study focuses on the surface coupling and strain-induced optoelectronic modulation on a few-layer VO_2_/MoS_2_/Si heterostructure. Figure 1b shows the band alignment of the optimized p–n–n–p-type structure with different Mo-O sputtering times to control the MoS_2_ bandgap^33^. The nanostructured thin layer of Mo_0.2_W_0.8_O_3_ was deposited on the surface of VO_2_ as a protective and anti-reflection layer^34^. The optical anti-reflective layer (Mo_0.2_W_0.8_O_3_) is used to improve the responsivity of the photodetector and strongly eliminate the optical interference to minimize its undesired effects. Numerous applications including photodiodes^35^, image sensor^36^, and semiconductor photodetectors^37^ have recorded the uses of the antireflective coating. The structural and morphological characteristics are also studied here. Raman, PL, electrical, optoelectronic characterization of strongly correlated oxide (VO_2_), and 2D VDW heterostructure (MoS_2_-Si) will be discussed.

## Results and discussion

### Structural and composition

**Figure 2:**
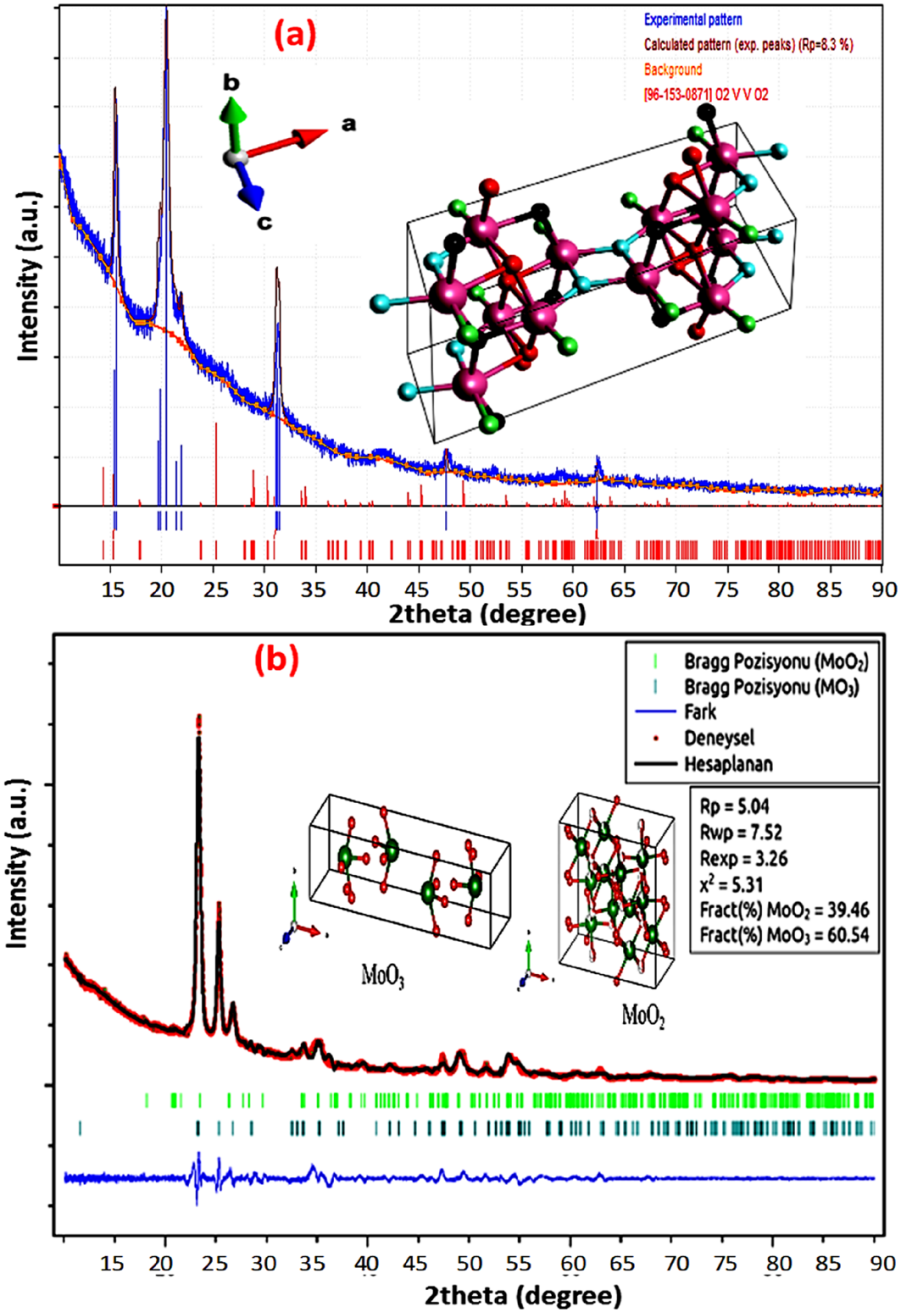
XRD crystal structure of (**a**) monoclinic VO_2_ and (**b**) Rietveld structure refinement XRD pattern of molybdenum oxide (in our case MoO_2_ and MoO_3_). The inserted 3D crystal structures are obtained using VESTA 3D visualization program (Model VESTA 3; https://jp-minerals.org/vesta/en/).

The crystal structure of vanadium oxide and molybdenum oxide was calculated in detail to understand the structural analysis of the deposited films. Figure 2a shows the XRD pattern of the deposited VO_2_ thin film. XRD result of VO_2_ shows a monoclinic phase with a JCPDS card number of [#96-153-0871] with a space group of C12/m_1_(12). The inserted crystal structure and visualizing 3D data are obtained using VESTA crystal software. Different diffraction peaks are observed at 2θ = 15.45°, 20.49°, 31.28°, 47.69°, and 62.32°. The unit cell parameters are a = 12.03000 Å, b = 3.69300 Å, c = 6.42000 Å while the angle is β = 106.100°. However, the Wyckoff position of metal atoms are (2a): (0, 0, 0) and (4f): (0.25, 0.25, 0.25) and oxygen atoms are (4i): (0.175, 0, 0.25), (0.175, 0.5, 0.25) and (8j): (0.075, 0.25, 0.75). The calculated amount of O:V in the VO_2_ compound indicating that more oxygen vacancies. Oxygen deficient in vanadium oxide (VO_2−δ_) has reported many times to stabilize the metallic state of VO_2_^38^, decrease the semiconductor–metal phase transition (SMT)^39^ as reported here and to narrow the bandgap in the monoclinic phase^40^. Oxygen vacancies are acting as an electron donor with n-type conductivity which can change the electron orbital occupancy, band structures, and contribute to high photocurrent gain when staking with MoS_2_^41^.

The preparation of molybdenum oxide was achieved using the Mo target at sputtering temperature of 400 °C. Figure 2b shows the XRD Rietveld refinement of Mo-O bonding in molybdenum oxide. The calculated R-factors were found to be R_wp_ = 7.52, R_exp_ = 3.26, and χ = 25.31. The Refinement analysis shows that MoO_3_ and MoO_2_ crystal phases are contributed. The distribution of atoms in unit cells of MoO_2_ and MoO_3_ was plotted with the VESTA program and shown in Fig. 2b. The amounts of Mo in MoO_2_ and MoO_3_ were calculated as 1.837 and 1, respectively. While the amounts of O_2_ in MoO_2_ and MoO_3_ were 3.836 and 3.012, respectively. The calculation of the crystal structure was showed that 9.46% as MoO_2_ phase and 54% as MoO_3_ phase. Moreover, the unit cell parameters of the MoO_2_ structure are a = 9.788, b = 8.604, c = 4.714 Å. While for MoO_3_ structure are a = 15.309, b = 3.719, c = 3.976 Å.

### Raman characterization

**Figure 3:**
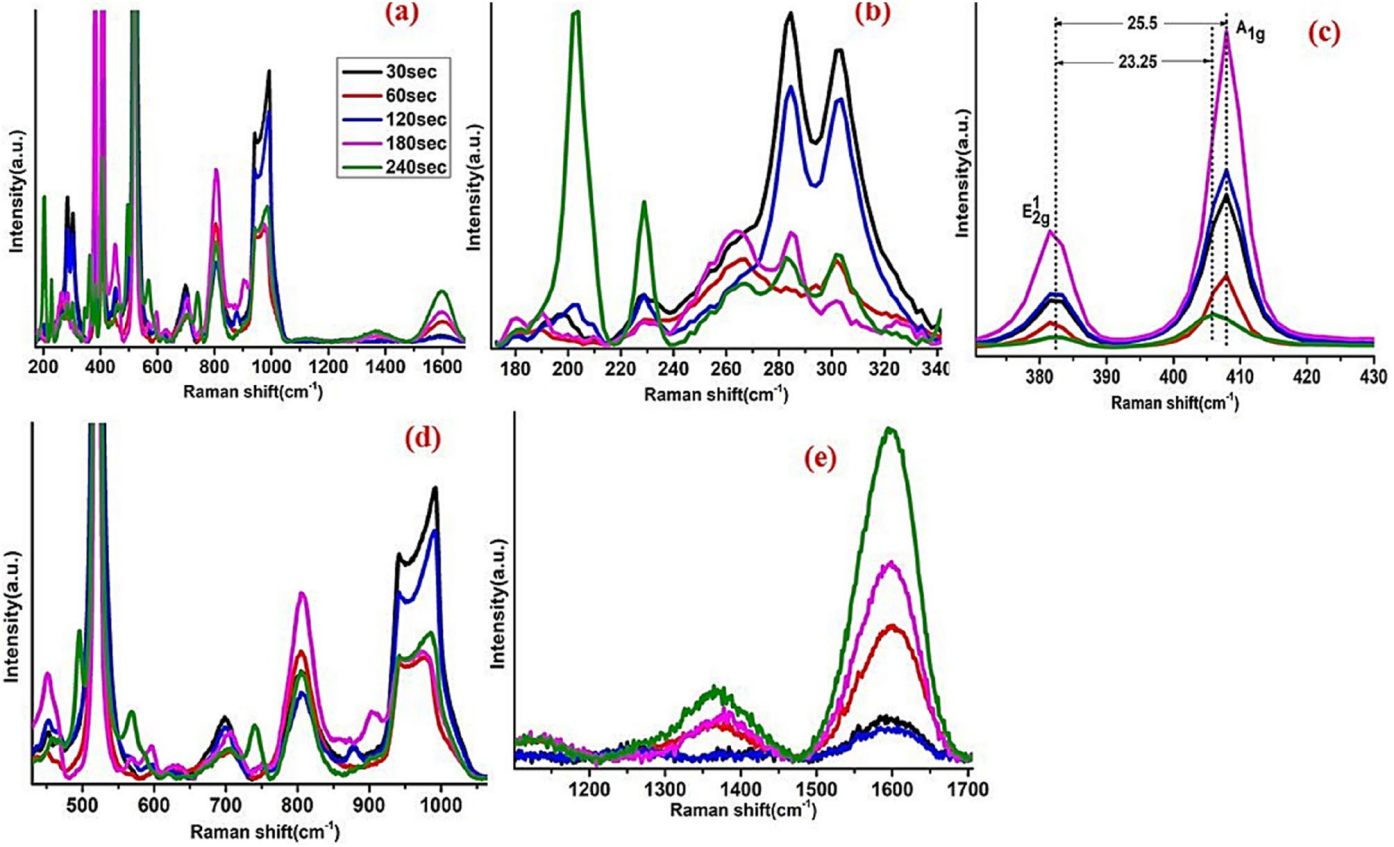
Raman spectra of the deposited multilayer MoWO_3_/VO_2_/MoS_2_ on p-type Si substrate; (**a**) the full range (200–1700 cm^−1^) Raman spectra of the deposited films; (**b**–**e**) magnified ranges of the full range spectra.

**Figure 4:**
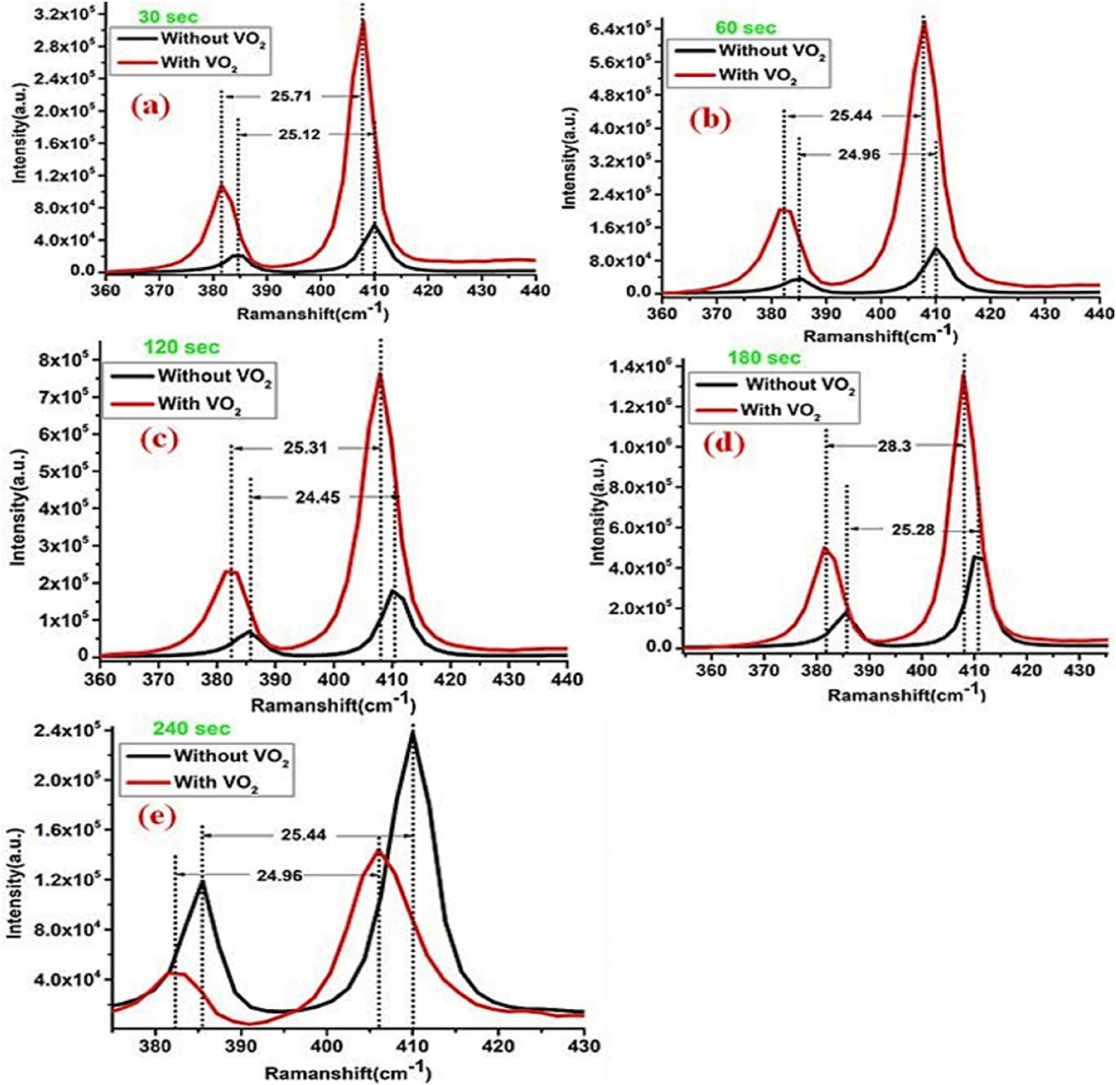
Raman peaks of MoS_2_/Si fabricated at different sputtering times in the presence and absence of VO_2_ layer on the surface.

The physics behind interfacing structures such as 2D semiconductors and correlated oxides should receive high attention. The importance of these structures can be highlighted by controlling the band alignment of the 2D materials such as MoS_2_. Moreover, controlling the carrier mobility, coupling, and strain effect (as reported in the current work)^42^. Raman spectra of the deposited multilayer structure MoWO_3_/VO_2_/MoS_2_ on p-type Si substrate are depicted in Fig. 3. The full range (200–1700 cm^−1^) Raman spectra of the deposited structures are shown in Fig. 3a and the magnified ranges of the full range spectra are shown in Fig. 3b–e. The Raman peaks of VO_2_ at RT are shown in Fig. 3a,b,d which confirm its monoclinic phase. Whereas, Fig. 3c shows the two characteristic peaks of MoS_2_ of ${E}_{2g}^{1}$ (385 cm^−1^) and ${A}_{1g}$ (405 cm^−1^) originate from their in-plane and out-of-plane phonon characteristics^43^. The peaks positions, intensities, distance between ${E}_{2g}^{1}$ and ${A}_{1g}$ position at different sputtering time of MoS_2_ are summarized in Table 1 and Fig. 4.

**Table 1:** The Raman peaks positions and intensities of VO_2_/MoS_2_/Si and MoS_2_/Si structures.

Deposition time	30 s		60 s		120 s		180 s		240 s	
VO_2_ layer	With	Without	With	Without	With	Without	With	Without	With	Without
Position of ${E}_{2g}^{1}$ (cm^−1^)	382.02	384.91	382.16	385.26	382.49	385.93	381.51	385.51	382.51	385.39
Position of ${A}_{1g}$ (cm^−1^)	407.73	410.03	407.61	410.22	407.80	410.39	409.86	410.80	406.14	409.79
Position difference (${E}_{2g}^{1}-{A}_{1g}$) (cm^−1^)	25.71	25.12	25.44	24.96	25.31	24.46	28.31	25.28	23.63	24.40
Intensity of ${E}_{2g}^{1}$	110,852.8	22,177.5	204,858.9	39,921.1	231,297.2	71,156.9	503,416.9	191,107.0	45,413.2	120,485.6
Intensity of ${A}_{1g}$	313,824.8	60,847.9	664,071.8	113,963.2	762,847.3	180,168.1	1,364,933.3	465,300.5	144,965.8	240,574.3
Intensity difference (${E}_{2g}^{1}-{A}_{1g}$)	202,972.0	38,670.4	459,212.9	74,042.1	531,550.1	109,011.2	861,516.4	274,193.5	99,552.6	120,088.7

Many authors tried to study the effect of VO_2_ in contact with transition metal dichalcogenides semiconductors (TMDs) layers such as MoS_2_ and WS_2_^31,32^. While in our current study, we tried to study the matching behavior of 50 nm thin VO_2_ layer onto the surface of MoS_2_/Si heterojunction. Additionally, the current work showed that different thicknesses of Mo-O structure have a direct effect on Raman, PL, electrical and optoelectronic characterization of MoS_2_ peaks (position and intensity). It is seen from Raman spectra that with incorporated the monoclinic VO_2_ layer, there is a redshift and an increase in the intensity peaks as in Fig. 4a–d. These observations showed that MoS_2_ and VO_2_ were electronically coupled, similar results observed before^32^. The observed shift in Raman modes may be attributed to the presence of compressive strain induced by implementing the VO_2_ layer, similar results observed when interfacing MoS_2_ with PMN-PT^44^. Generally, compression stress exerted on VDWs structure decreases the lattice constant^44,45^ and consequently increases the film crystallinity and photocurrent as reported here.

It is known that the intensity of Raman peaks is referring to high crystallinity effects. In most cases, Raman scattering is sensitive to the degree of crystallinity in a sample. Typically a crystalline material yields a spectrum with very sharp, intense Raman peaks, whilst an amorphous material will show less intense Raman peaks^46–48^. Table 1 and Fig. 4 show the Raman intensities of the corresponding thin films. These results show high attention to the applications of enhancing the Raman signal/intensity. The difference between peak positions of ${E}_{2g}^{1}$ and ${A}_{1g}$ in the presence and absence of VO_2_ layer are deposited in Table 1. It shows a decrease with increasing the MoS_2_ layer thickness from 30 to 240 s for both MoS_2_/Si and VO_2_/MoS_2_/Si structures, which may contribute to a decrease in the film layers and enhance the band gap^49^ as seen from Table 1. However, the film deposited at 180 s is out of this base. Meanwhile, the differences between the intensities of the peaks show an enhancement in the peak intensity with increasing the MoS_2_ film thickness which may contribute to high crystallinity effects. In counter, the film deposited at 240 s shows a decrease in the intensity. The highest intensity was observed for VO_2_/MoS_2_/Si with 180 s, whereas the lowest intensity was attributed to 240 s film. These results are concluded that the MoS_2_ sputtering time of 180 s is optimized for VO_2_ and MoS_2_ optical coupling. Consequently, the observed results may highlight the importance of incorporating strongly correlated oxide through 2D VDWs MoS_2_ structure to control film crystallinity, surface-enhanced Raman spectroscopy (SERS) of MoS_2_ for better signal detection and spatial resolution^50–52^, optical coupling^53,54^, Plasmonic local-field enhancement^53^ and optoelectronic behavior^51^.

For more details about intensity distribution versus position, Raman mappings of ${E}_{2g}^{1}, {A}_{1g}$ and Si peaks of MoS_2_/Si at different sputtering time of Mo-O (30, 60, 120, 180, and 240 s) are investigated at 385, 410, and 520 cm^−1^, respectively, and shown in Fig. S3 (a, b, c, d, and e). The Raman mapping was realized at large area (1.6 mm × 1 mm) with 320 × 200 data point using Leica microscope at 5 × magnification to provide an evidence about the ability to scale up the MoS_2_ thin films. The wavelength, power and integration time of the used laser were 532 nm, 3 mW and 1 s, respectively. This analysis illustrated that 30, 60, and 120 s samples show high homogeneity over the full scale (1.6 × 1 mm^2^). Although, 180 s sample shows regular agglomerations of nanoparticles for both E_2g_ and A_1g_ positions, the 240 s sample shows irregular clusters. The scaling up of MoS_2_ can therefore be demonstrated until sputtering time < 180 min.

### Photoluminescence measurements (PL)

**Figure 5:**
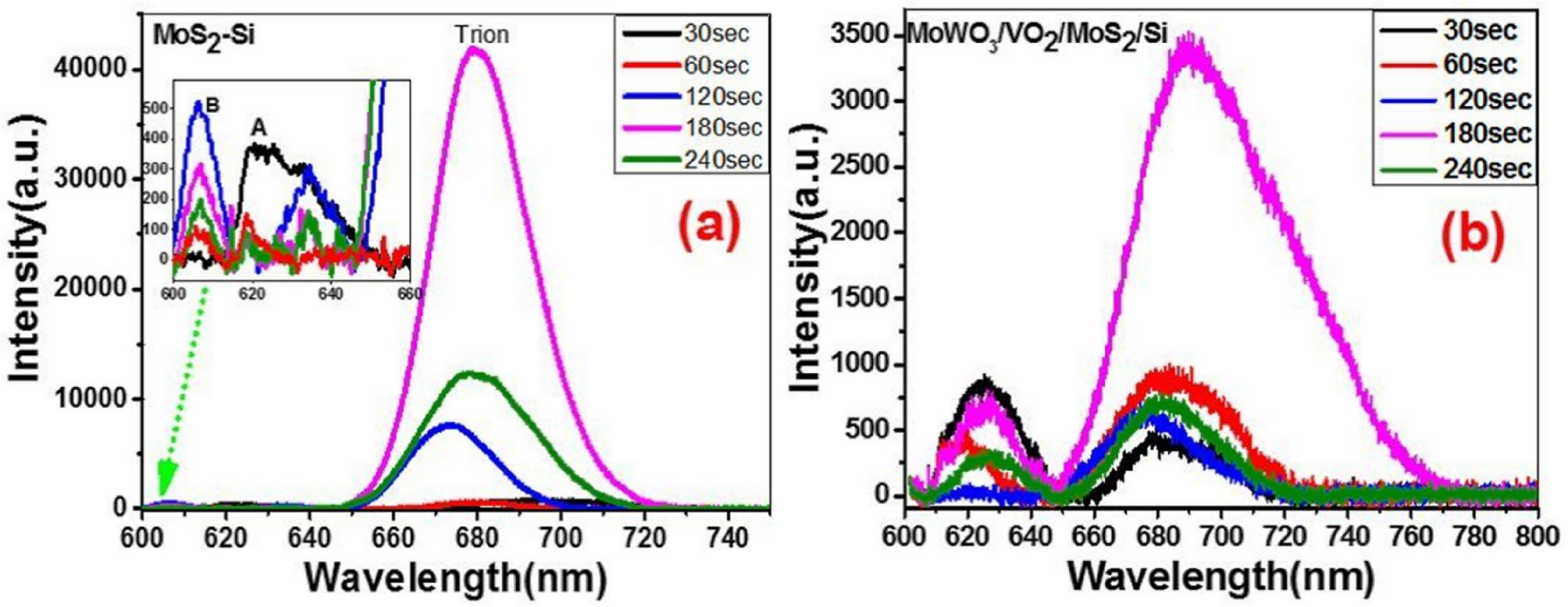
PL spectra of (**a**) MoS_2_-Si heterojunction and (**b**) MoWO_3_/VO_2_/MoS_2_-Si photodetectors.

**Figure 6:**
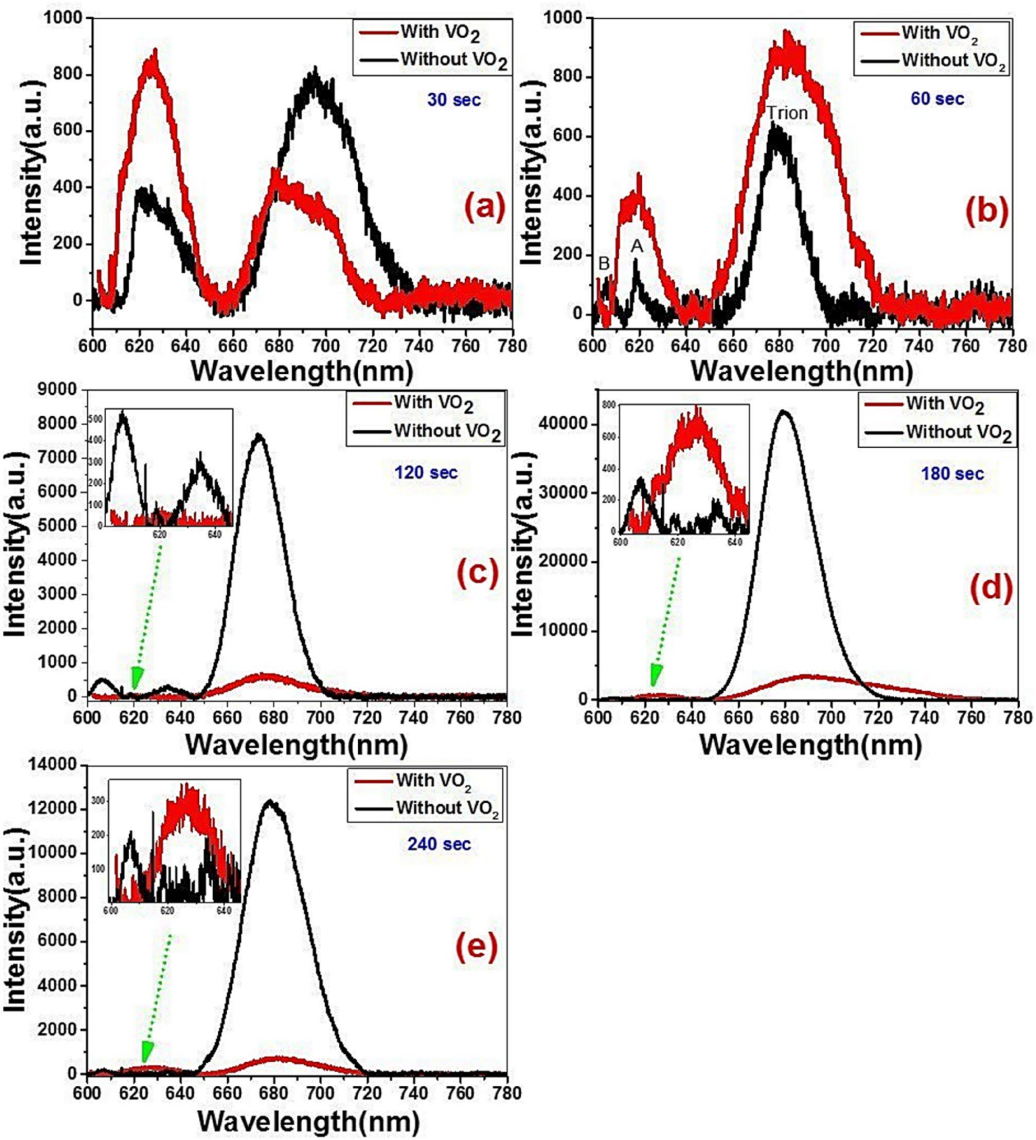
PL spectra of MoS_2_/Si fabricated at different sputtering times without and with VO_2_ supporting layer on the surface.

The PL spectrum of MoS_2_ is strongly dependent on the number of layers of 2D VDWs structures. In other words, a strong PL peak may observe in single-layer MoS_2_ or WS_2_ film and decreasing with increasing the number of layers^55^. Trion is defined as a quasi-particle that can potentially carry out more information and data than electrons for which make them useful towards different applications such as optoelectronics and quantum computing^56^. Trions are consisting of three charged particles bound together by very weak bonding energy that makes them quickly fall apart^57^. It is known that the dominated peak in Figs. 5 and 6 attributed to the recombination of the photogenerated electron–hole pair, whereas the observed weaker peak at lower wavelength may be attributed to the valance band splitting due to the presence of strong spin–orbit coupling of MoS_2_^58^. In the literature review, the definition of trion and exciton peak is dominated by their locations and the trion peak is located at lower energy than exciton peak^59–61^. Figure 5a and b shows the PL spectra of MoS_2_/Si and MoWO_3_/VO_2_/MoS_2_/Si structures at RT. The MoS_2_/Si has two peaks at ~ 679 and ~ 620 nm which corresponding to components from trion and A-exciton^62^. However, trion and A-exciton positions have changed by controlling the deposition time of MoS_2_ as seen in Table 2. Also, this table shows the position of the characteristic peaks (A-exciton, B-exciton (higher spin–orbit splitting state), and trion) in the presence and absence of the VO_2_ layer. Generally, it is seen that the trion peak shifted to lower energy by implementing the VO_2_ layer onto the surface of the MoS_2_/Si structure as seen in Table 2.

**Table 2:** The PL peaks positions and intensities of VO_2_/MoS_2_/Si and MoS_2_/Si structures.

MoO_3_ deposition time	30 s		60 s		120 s		180 s		240 s	
VO_2_ layer	With	Without	With	Without	With	Without	With	Without	With	Without
**Peak position (nm)**										
Trion	678.65	696.06 Blue shift	682.47	679.32 Red shift	676.29	673.08 Red shift	690.40	679.54 Red shift	681.32	678.78 Red shift
A-exciton	625.08	622.21 Red shift	617.20	618.11 Blue shift	602.82	634.7 Blue shift	624.97	633.50 Blue shift	626.15	635.12 Blue shift
B- exciton				606.80		605.69		606.41		606.69
**Intensity**										
Trion	425.54	782.63	939.08	609.64	621.29	7554.96	3383.10	41,642.54	712.12	12,328.14
A-exciton	955.40	378.21	401.69	136.24	64.85	295.21	658.62	134.53	292.37	145.70
B-exciton				88.73		514.81		300.94		209.37

The A-exciton peak has a higher intensity in the case of the VO_2_/MoS_2_/Si structure than in the case of MoS_2_/Si structure for 30 and 60 s deposition time of MoS_2_ layer, Fig. 6a and b. The trion peak is enhanced and shifted to longer wavelengths by increasing the deposition time to 60 s. The increase in PL intensity refers to an enhancement in light emission efficiency and increases the density of states of the photo carriers by modifying the band structure^44^. At 30 s, the A-exciton peak intensity is higher for the VO_2_/MoS_2_/Si structure than the MoS_2_/Si structure. i.e., the incorporation of VO_2_ dramatically changes the ratio between photoluminescence intensities of A-exciton and trion from < 1 to > 1 for VO_2_/(30 s)MoS_2_/Si structure. However, the opposite case is observed for the trion. Meanwhile, by incorporating the VO_2_ layer, a blue shift in the trion peak is observed, while a redshift is observed for the A-excitons. The observed peak position of PL that shifted towards lower energy (redshift) attributed to the non-radiative electron–hole recombination effect. However, by increasing the deposition time to 60 s, the A-exciton peak have higher intensity compared with the trions peak in the case of VO_2_/MoS_2_/Si structure than MoS_2_/Si structure as seen in Fig. 6b. By increasing the deposition time of MoS_2_ from 60 to 180 s, the B-exciton/A-exciton relative intensity increases, whereas the lowest ratio at 60 s deposition time refers to the high quality and the low defects densities of VO_2_/(60 s) MoS_2_/Si structure. Moreover, a redshift was observed for the trion peak and a slight blue shift when incorporating the VO_2_ layer. The increase in PL intensity refers to an enhancement in light emission efficiency and increases the density of states of the carriers by modifying the band structure and consequently enhance the radiative recombination of carriers, similar results were observed in a compressively strained trilayer MoS_2_ sheet^44,63,64^. This result shows that strong coupling between VO_2_ and MoS_2_ at 60 s Mo-O deposition time was observed at room temperature. While the PL intensity with MoS_2_/VO_2_ structure has only enhanced with increasing the film temperature^31,32,65^, while our reported results show a dramatic enhancement in the PL intensity at RT by incorporating VO_2_ layer on the surface of MoS_2_/Si structure.

On the other hand, trion peak quenching was found in Fig. 6c,d,e; with increasing the Mo-O layer from 120, 180, and 240 s when VO_2_ is deposited on MoS_2_/Si structure. We thought that the quenching of PL spectra in Fig. 6c,d,e may be owing to the fact that MoS_2_ is an n-type with a close Fermi level to the conduction band. However, the deposited VO_2_ layer at thicker Mo-O layer (120, 180, and 240 s) may shift Fermi level to the mid-band gap by drawback the electron coupling of VO_2_ and MoS_2_^66,67^. Similar results observed using back-gating with SiO_2_/Si^66^, dopants molecule like F4-TCNQ, metal-centered Phthalocyanine molecules on the surface of monolayered TMD^55,68^. It is interesting to note that in all Fig. 6a–e, A-excitons have higher intensities when incorporated VO_2_ layer. This result draws high attention for enhancing A-exciton peak intensity and raises strong spin–orbit coupling by incorporating the monoclinic VO_2_ thin layer. Moreover, the B-exciton peak was observed in the MoS_2_/Si structure at 606 nm (2.04 eV), but it did not appear in the VO_2_/MoS_2_/Si structure as seen in the insets of Fig. 6b–e. It is known that the PL spectra of MoS_2_, surprisingly, increases with decreasing layer thickness^62^. However, the origin of PL spectrum in MoS_2_ arises from the direct excitonic electronic transitions which shows higher radiative recombination rate than nanocrystals^69^. Therefore, the enhanced photoluminescence with increasing the deposition time of Mo-O has to be attributed to a dramatically slower electronic relaxation factor ${\kappa }_{relax}$ as in Eq. 1, suggesting a substantial change in electronic structure of MoS_2_ when going from the short to longer deposition time of Mo-O as seen in Fig. 6.

1 $$\eta _{{Lum}} \,\sim\,\frac{{\kappa _{{rad}} }}{{(\kappa _{{rad}} + \kappa _{{defect}} + \kappa _{{relax}} )}}$$

where ${\kappa }_{rad}, {\kappa }_{defect}, {\kappa }_{relax}, and\,\,{ \eta }_{Lum}$ are representing the rates of radiative recombination, defect trapping, and electron relaxation, Luminescence quantum efficiency within the conduction and valence bands, respectively.

It is concluded that by depositing the VO_2_ layer on MoS_2_/Si structure, both trion and exciton peaks get shifted as seen in Table 2. It is implemented that the presence of the VO_2_ layer on the surface of the MoS_2_/Si structure results in a redshift through trion peaks, while a blue shift for A-exciton. The peak position of PL for trion is shifted towards lower energy due to the occurred non-radiative electron–hole recombination. However, 30 s sample is out of this rule with a blue and redshift in the trion and A-exciton peak on the VO_2_ layer, respectively.

### Surface topography and grain boundary mapping

**Figure 7:**
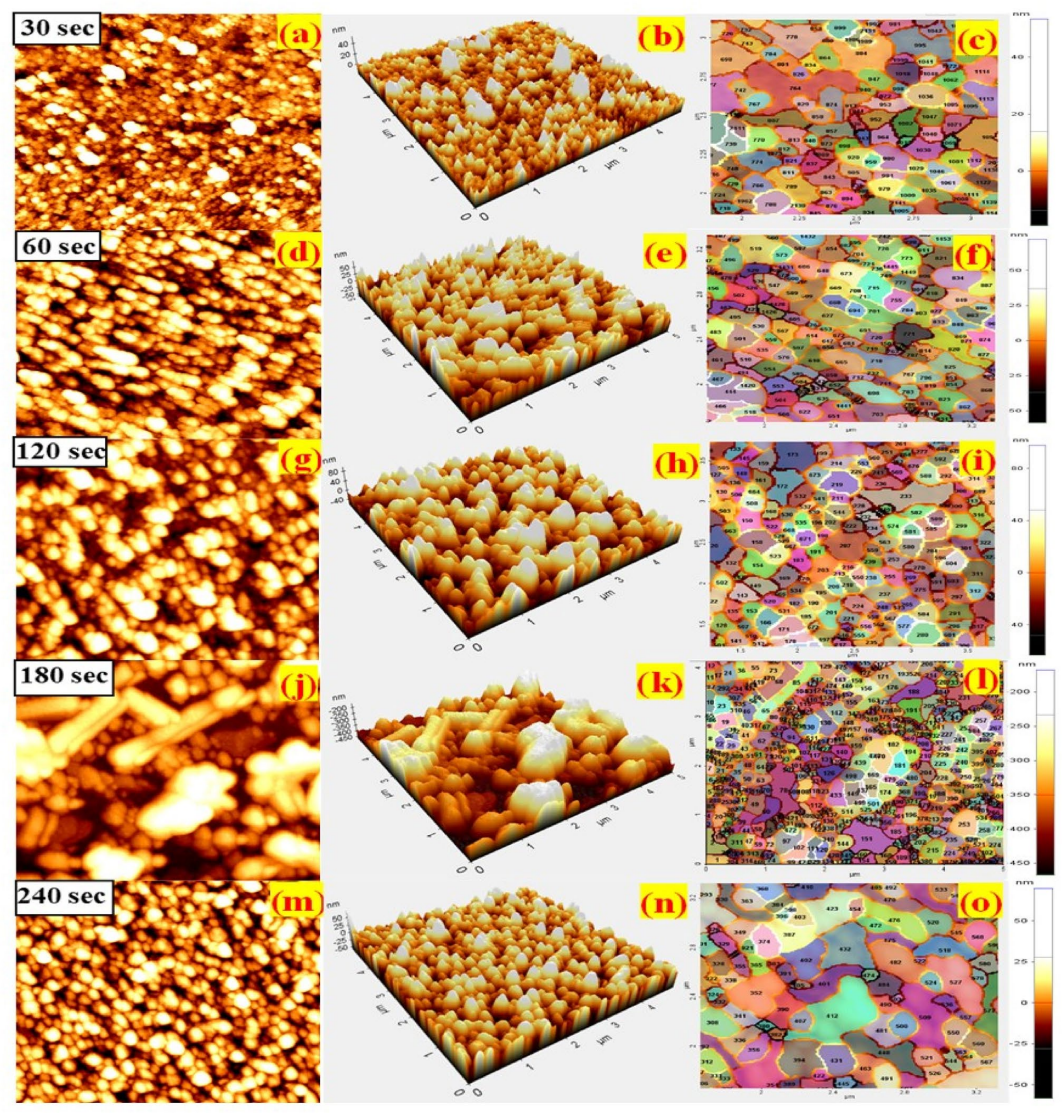
2D AFM images (**a**, **d**, **g**, **j**, and **m**), 3D AFM images (**b**, **e**, **h**, **k**, and **n**), and the grain/grain boundary mapping (**c**, **f**, **I**, **l**, and **o**) of the prepared MoWO_3_/VO_2_/MoS_2_-Si thin film with sputtering time of 30, 60, 120, 180, and 240 s, respectively. XEI software was used for image analysis and processing. [XEI PSIA, Version 1.5, https://parksystems.com/102-products/park-xe-bio].

AFM has been used to investigate the surface topography, roughness, grain, and grain boundary mapping. Surface topography and parameters such as average roughness R_a_(nm), root mean square or standard deviation of the height value R_q_(nm), height different or peak-to-valley (R_pv_), ten-point height (R_z_), skewness (R_sk_) and kurtosis (R_ku_) as well as fractal and grain analysis were inspected by the XEI software. Figure 7a,d,g,j,m shows a 2D surface topography, Fig. 7b,e,h,k,n shows the 3D visualization, and Fig. 7c,f,I,l,o shows the grain boundary mapping of the prepared MoWO_3_/VO_2_/MoS_2_/Si thin film with sputtering time of 30, 60, 120, 180, and 240 s, respectively. The films that deposited at short deposition time show higher uniformity, while with increasing the deposition time a small clusters of different sizes less than 100 nm have been observed. The average roughness values of the prepared thin films have summarized in Table 3 and show that 30 s and 180 s thin films have the lowest and highest R_a_ value of 3.28 and 48.0 nm, respectively. It seems that with increasing the deposition time of Mo-O, the accumulated nanoparticles show bigger sizes, consequently higher roughness factors. The calculation of the grain and grain boundaries of interfacing thin films are important parameters that provided information about the nature of interfaces between two layers. Figure 7c,f,I,l,o shows the grain size and grain boundary distribution maps of the prepared thin films. The SEM images of VO_2_, MoO_3_, Mo_0.2_W_0.8_O_3,_ and MoS_2_/Si thin films are presented in Figures S1 and S2 and discussed in supplementary information. Large scale MoS_2_ thin films have been studied in our previous work by combining CVD and sputtering technique^70^. SEM images (Fig. S2, supplementary information) are provided to illustrate a homogenous and approximately uniform nanoparticles distribution. The SEM images in Fig. S2 (supplementary information) are provided to illustrate the homogeneity and approximate uniform surface distribution of nanoparticles, particularly for sputtering time < 180 s. Also, the AFM images of the MoWO_3_/VO_2_/MoS_2_-Si thin films, Fig. 7, show almost uniformly distributed grains for sputtering time up to 120 s. Besides the presented grain/grain boundaries mapping in Fig. 7, the scanning area of the AFM images was selected to be 5 µm × 5 µm to provide evidence about the ability to scale up the MoS_2_ thin films.

**Table 3:** The roughness parameters of MoWO_3_/VO_2_/MoS_2_/Si photodetector.

Parameters	30 s	60 s	120 s	180 s	240 s
R_pv_ (nm)	26.364	76.913	91.878	226.585	73.849
R_q_ (nm)	4.458	15.987	22.246	55.048	12.934
R_a_ (nm)	3.283	13.460	18.430	48.080	10.688
R_z_ (nm)	22.126	59.141	74.573	161.619	48.946
R_sk_	− 1.143	0.026	− 0.196	− 0.172	− 0.091
R_ku_	5.362	2.333	2.143	1.973	2.895

### Temperature-resistance measurement (T-R)

**Figure 8:**
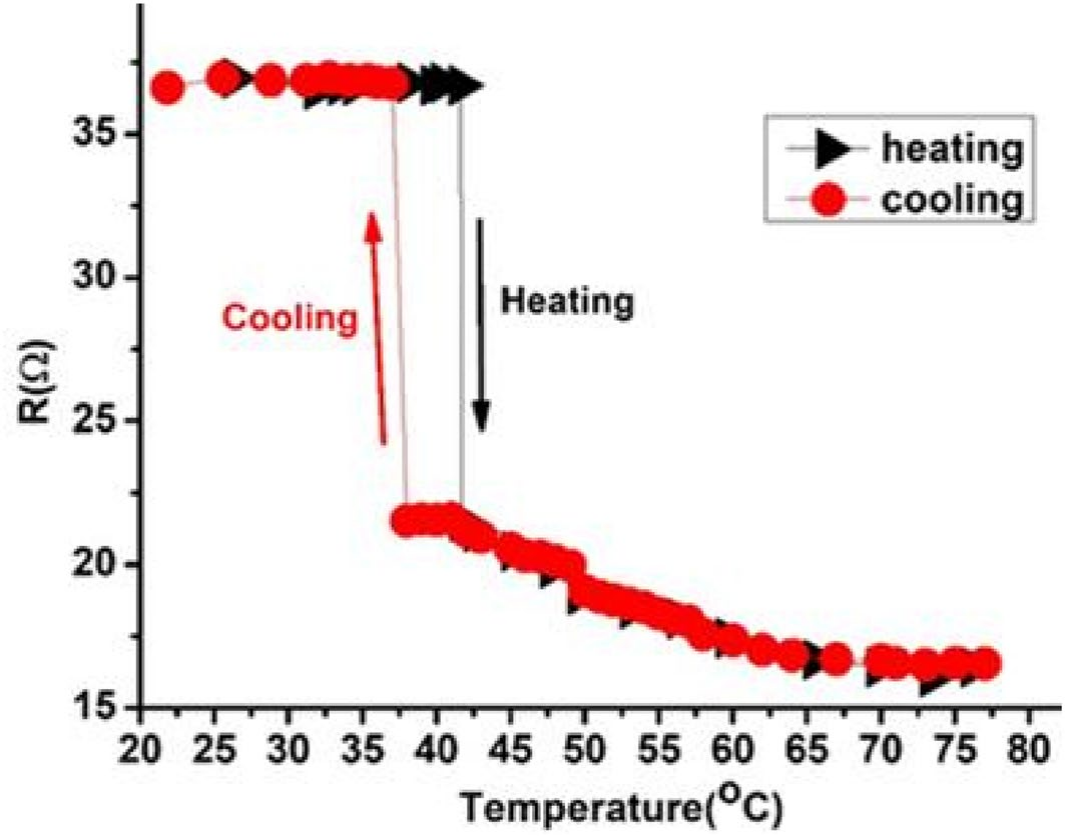
Semiconductor–metal phase transition of 50 nm thin VO_2_ layer from room temperature to 78 °C.

The phase transition of the prepared VO_2_ thin film has been performed using a four-probe measurement system connected to a heating stage ranging from RT to 78 °C. We investigated the influence of the 50 nm VO_2_ thin layer on Raman, PL, and optoelectronic measurements of a few-layers MoS_2_ with different sputtering times of Mo-O layer. So, the electrical semiconductor–metal phase change of VO_2_ has been tested as depicted in Fig. 8. In our case, the VO_2_ phase transition temperature was calculated to be 40 °C by controlling the sputtering condition (high vacuum and long-time annealing temperature) which may affect the lattice-strain and oxygen vacancy concentrations of VO_2_^71–73^. The reason for the low semiconductor-metallic phase transition may owing to the high concentration of oxygen vacancies. Oxygen deficient in vanadium oxide (VO_2−δ_) has reported many times to stabilize its metallic state^10^, decrease the semiconductor–metal transition (SMT)^11^ and narrowing the bandgap of the monoclinic VO_2_ phase^12^. Oxygen vacancies are electron donors with n-type conductivity which can change the electron orbital occupancy and band structures and contribute to the high photocurrent generation when staking by MoS_2_^13^. To check these coupling effects between theses layer, UV-optoelectronic measurements have been carried out.

### Electric and optoelectronic properties

**Figure 9:**
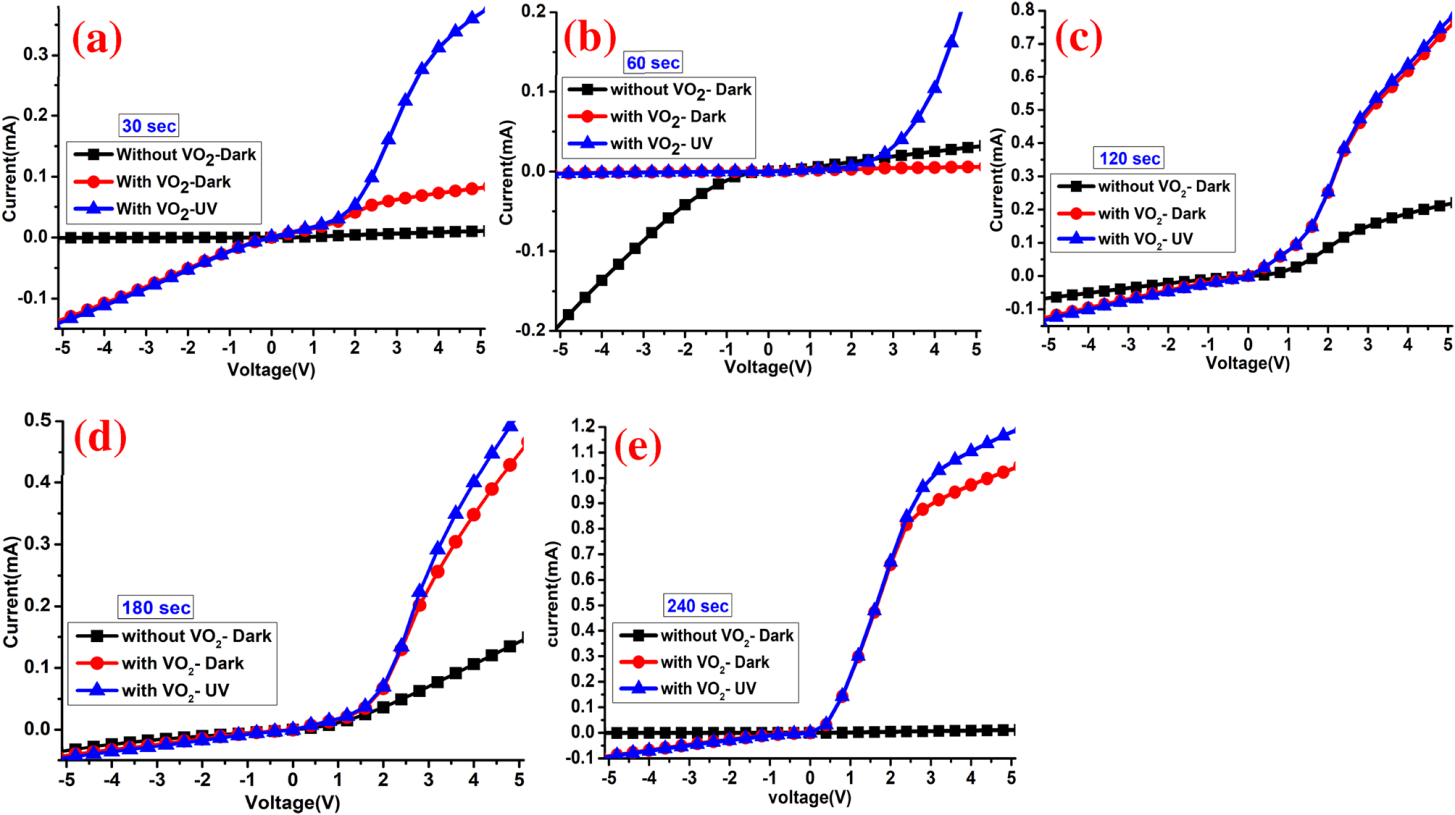
The linear I–V characteristics of the MoWO_3_/VO_2_/MoS_2_/Si device in dark and under UV illumination with MoS_2_ sputtering time of (**a**) 30, (**b**) 60, (**c**) 120, (**d**) 180, and (**e**) 240 s.

This section discusses the electric characterization of MoS_2_/Si heterostructures before and after depositing the VO_2_ layer under dark and UV conditions. In order to investigate the I–V and photoresponse of the prepared devices, we measured the I–V curve under dark and upon UV light illumination by applying a sweep voltage from − 5 to + 5 V for different sputtering times of Mo-O as shown in Fig. 9. This figure shows the electrical and optoelectronic properties of MoWO_3_/VO_2_/MoS_2_/Si thin film with different thicknesses of the MoS_2_ layer. Figure 9a–e shows the current–voltage (I–V) curves under dark and UV illumination at MoS_2_ deposition time of 30, 60, 120, 180, and 240 s, respectively. The observed photocurrent in this figure is larger than that reported for previously proposed MoS photodetector with lateral contacts arrangement^31^. The back Al and front Pd-Au contacts may paly important rule in that because these contacts allow the vertical electron transport in the heterostructure photodetector besides the lateral electron transportation and consequently 2D conductivity measurements^31,37^. The vertical electron transport offers a high density of active edge sites^37^. Also, our optimized heterojunction photodetector does not have a high-resistance layer like the SiO_2_ layer that was previously used and obstruct the vertical electron transport^31^. The back Al contact has been used for better collecting signals. Al metal makes Ohmic contact type with p-Si, which is also observed when probed on two contact pads on the same side, however, noble metals such as Ag, Au, etc. make Schottky contact with p-Si. On the other hand, the Au–Pd front contact was built in the anti-reflection Mo_0.2_W_0.8_O_3_ layer, in which the formed Schottky barrier height and width could be controlled by the current passing through the metal–semiconductor contact. Under dark conditions and as predicted by Basyooni et al., a non-linear I–V curve was obtained indicating that a good double-Schottky contact behavior was formed between the front Pd-Au contact and the film surface^34^. The position of asymmetric metal contacts can provide an integrated potential gradient assigned to the work function difference of asymmetric electrodes as previously stated for various applications, such as gas sensors^34^ and photodetector^74^, which leads to enhanced device performance as reported here. For instance, Casalino et al. used an asymmetric Al–Si–Cu (metal–semiconductor–metal) structure-based Si photodetector^75^. Moreover, several studies used asymmetric metal contacts for the photodetector application to control the dark current^70,74^.

Figure S4 shows the linear and semi-logarithmic scale current–voltage characteristics of MoS_2_/Si device without VO_2_ layer under the dark condition with different sputtering times of MoS_2_ layer; 30, 60, 120, 180, and 240 s. The positive part shows an increase in the associated dark current with increasing the sputtering time from 30 to 60 s. While at 120 s, a jump in the forward dark current is observed due to the related folding-effects in MoS_2_. Folding effect decreases the interlayer coupling and enhances the photoluminescence emission yield of A- and B-exciton peaks as seen in Fig. 5. Whereas instability measurements were observed at the negative bias part. The large increase in the negative dark current for MoS_2_(60 s) may be attributed to the release of charges that were trapped on MoS2 's surface at the interface trap sites (oxygen sites). The highest reverse dark current, which suggests the lowest potential barrier, was observed at 60 s. This may be ascribed to the values of the optical band gaps as shown in our previous study, whereas the 60 s MoS_2_-Si thin film displayed optical band gaps of 1.75 and 2.01 eV^33^. By increasing the sputtering time the reverse dark current decreases and almost becomes identical for sputtering time ≥ 180 s, as shown in Fig. S4(a,b).

**Figure 10:**
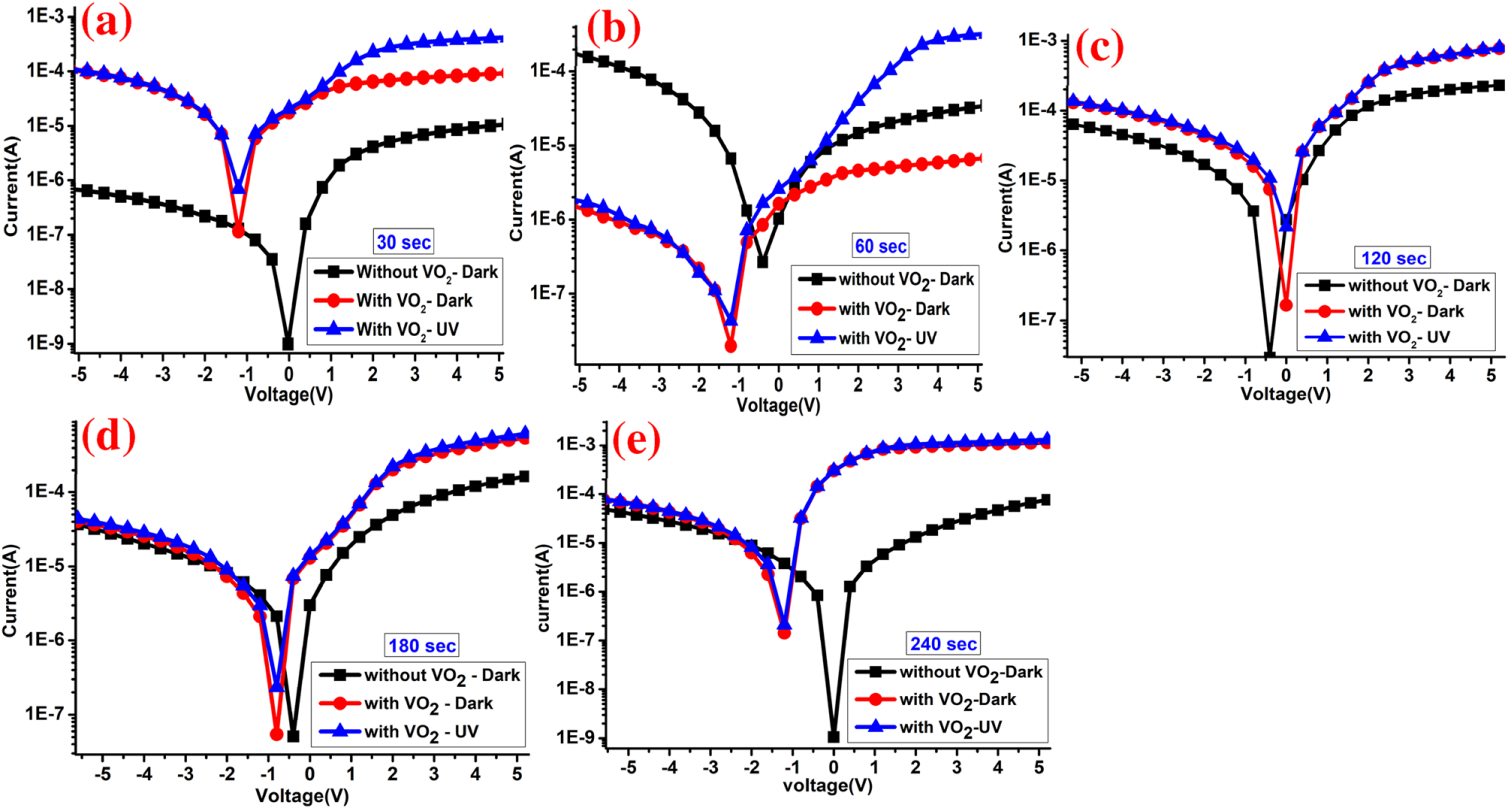
The semi-logarithmic scale I–V of MoWO_3_/VO_2_/MoS_2_/Si device under dark and UV illumination with MoS_2_ sputtering time of (**a**) 30, (**b**) 60, (**c**) 120, (**d**) 180, and (**e**) 240 s.

Figure 10a–e shows the log-current curves under dark and UV illumination of 30, 60, 120, 180, and 240 s conditions, respectively. It is clearly seen that UV illumination shifts the logarithmic I–V curve towards the negative voltage region. This behavior may address the induced strain effects from the VO_2_ layer or unidirectional charge transport mechanism from the top to bottom layer due to the different electron concentrations^76^. Interestingly, it seems that the VO_2_ layer enhances the positive and negative current. Meanwhile, the dark current obtained after depositing the VO_2_ layer on the surface of MoS_2_/Si is about 2–3 folds’ improvement over pure MoS_2_/Si device for 30 and 60 s samples, as in Fig. 10a,b. The observed higher value of photocurrent under UV illumination is also attributed to enhancement though the band-to-band excitation in the VO_2_/MoS_2_/Si region. Moreover, carrier recombination and tunneling across the device junction may be addressed as a reason for the enhancement I–V under UV illumination. Nevertheless, fast response and recovery times, high responsivity, high reliability, and low signal-to-noise ratio are important characteristics for detector applications^77,78^, which is discussed below in detail.

### Transient response

**Figure 11:**
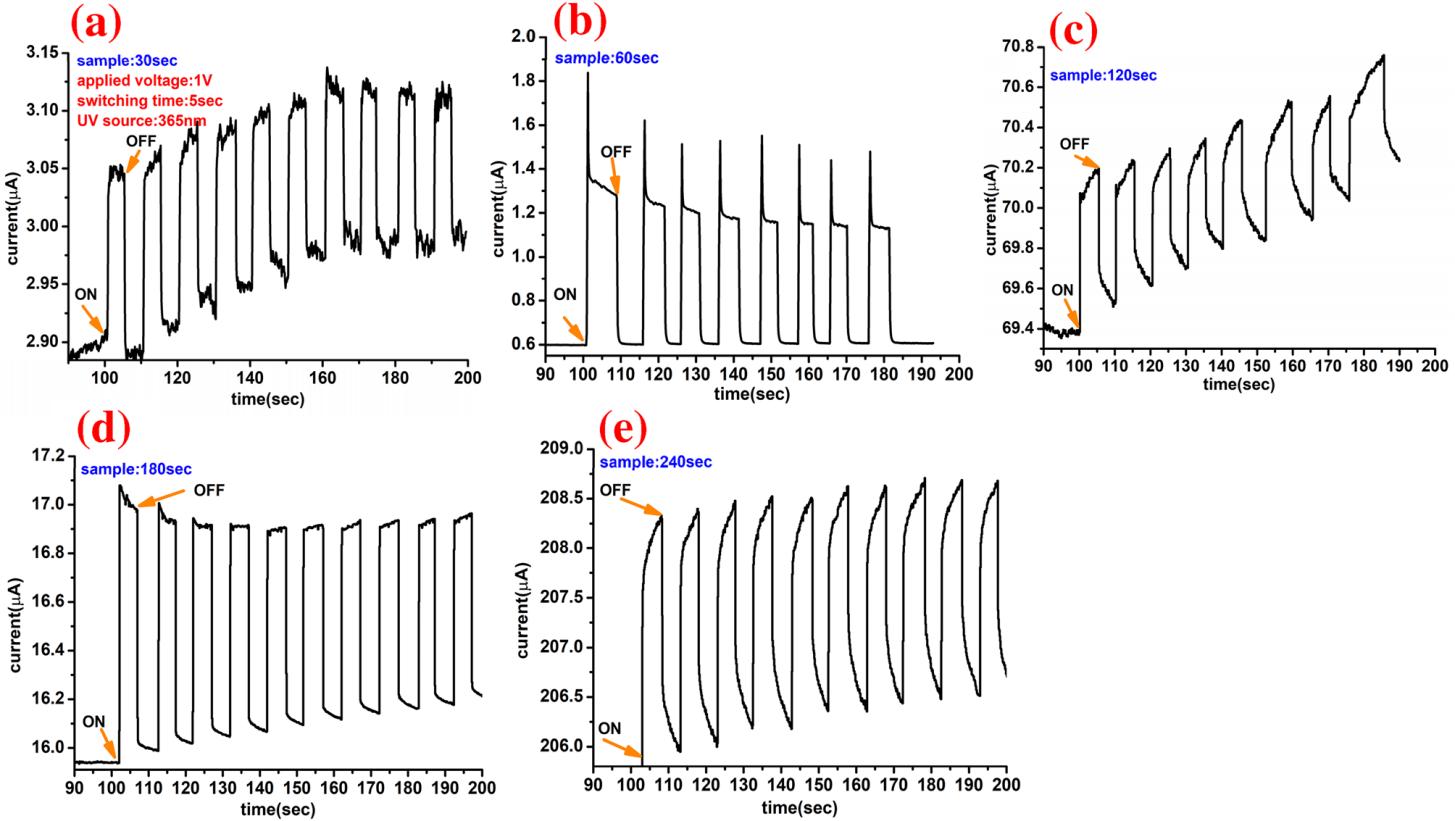
ON/OFF time-resolved photoresponse of the MoWO_3_/VO_2_/MoS_2_/Si device with MoS_2_ sputtering time of (**a**) 30, (**b**) 60, (**c**) 120, (**d**) 180, and (**e**) 240 s. All measurements were carried out at a bias voltage of 1 V, UV illumination of 365 nm, and witching time of 5 s.

Figure 11a–e shows the ON–OFF time-resolved photoresponses of MoWO_3_/VO_2_/MoS_2_/Si devices with varying MoS_2_ sputtering time (30, 60, 120, 180, and 240 s) in dark and under UV illumination. These optoelectronic transient/dynamic curves were measured with a switching time of 5 s, a bias voltage of 1 V, and UV illumination of 365 nm. Also, the optical modulations (photocurrent-time characteristics@ 1 V) under dark and UV illuminations of Mo_0.2_W_0.8_O_3_/VO_2_/MoS_2_/Si devices are presented in Figure S5 (supplementary information). Note that the ON/OFF and OFF/ON transitions of the UV light source are repeated many times for each 5 s at a bias voltage of 1 V. The time-resolved photoresponse curves in Fig. 11 show different sputtering time dependence. The curves at 30 and 120 s show increasing on/off behaviors, whereas the curves at 60, 180, and 240 s show stable on/off behaviors. The increasing on/off behaviors in Fig. 11a,c may come from some organic trap states that accumulated during the CVD sulfurization process. Wile, the stable on/off behaviors in Fig. 11b,d,e can be attributed to the high stability, high quality, and low density of the defects.

**Figure 12:**
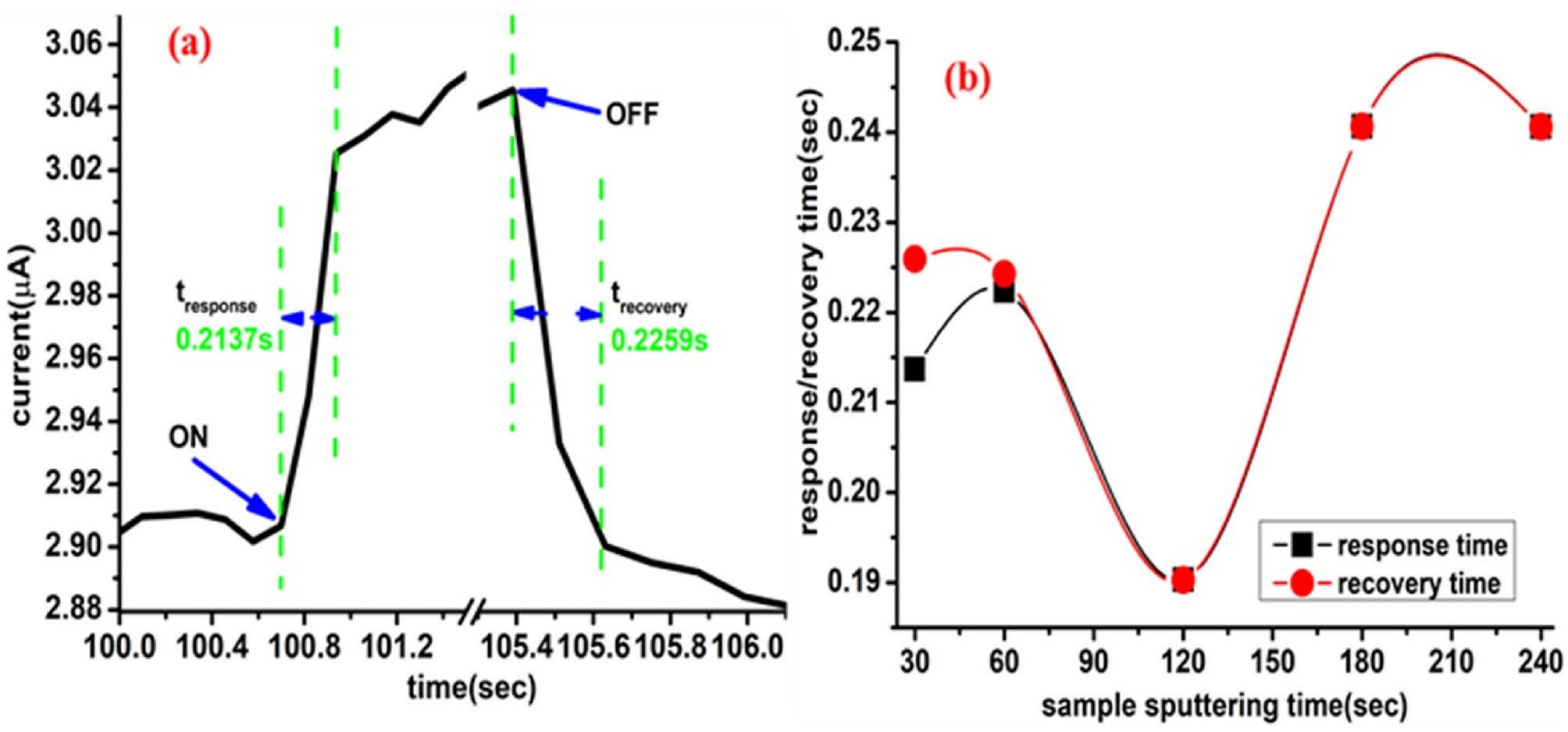
(**a**) Enlarged view of a single current–time photoresponse cycle for MoWO_3_/VO_2_/MoS_2_ (30 s)/Si UV photodetector to identify the response and recovery times and (**b**) the estimated response and recovery times as a function of the sputtering time.

The response/raise time was measured when the light source turned on, while the recovery/decay time was measured when the light turned off as shown in Fig. 12a. The response and recovery times have been estimated from the ON/OFF dynamic photoresponses at different sputtering times, Fig. 11. The combined sputtering and CVD deposition process of MoWO_3_/VO_2_/MoS_2_/Si UV photodetector device shows symmetrical response and recovery time which not exceed 0.25 s using the selected wavelength (365 nm) and bias (1 V), as shown in Fig. 12b. Consequently, our proposed photodetector is considered more efficient than the previously tested photodetector by Ang et al.^78^.

The fast response and recovery speed, indicating that electron–hole pairs could be effectively generated and separated in the proposed structure under UV illumination at room temperature. It is important to note that the fabricated device using MoS_2_(120 s) shows the fastest response/recovery times (0.19 s at 1 V) among the studied devices as shown in Fig. 12b. The fast response/recovery time at 120 s can indicate the fast and stable generation and separation of the electron–hole pairs. Unlike Dhyani and Das who reported rapid response for the silicon-MoS_2_ photodetector@ 580 nm and 3 V bias^79^, our measurements are performed without any external series resistance. Nevertheless, it is known that higher applied bias voltage can generate more photocurrent and consequently decrease the response and recovery time. A clear high photocurrent can be observed in the ON state at 1 V which makes the gate voltage lowers the potential barriers at the contacts, resulting in highly efficient photogenerated carrier extraction and thus increased photocurrent at a low applied voltage (1 V). The reason behind this is that the gate voltage can affect the height of the Schottky barrier between the metal contact and film surface and thus shift the Fermi level^80,81^. It seems that our designed photodetector did not require high bias voltage which makes it more applicable for low power photodetector technology.

### Photocurrent gain (Pg) and photoresponsivity (${\mathrm{R}}_{\uplambda }$)

The induced photocurrent I_ph_ is given by ${I}_{ph}={I}_{Light}-{I}_{Dark}$, where ${I}_{ph}$ increases with increasing the applied voltage and the light power^82^. Photocurrent gain (P_g_) can be defined and determined by ${P}_{g}=({I}_{photo}-{I}_{dark})/{I}_{dark}$, where ${I}_{photo}$ and ${I}_{dark}$ are photocurrent and dark current respectively^77^.

**Figure 13:**
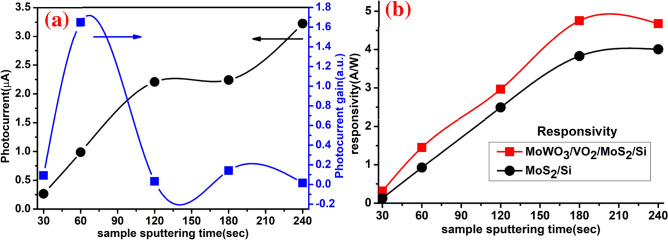
(**a**) the photocurrent and photocurrent gain of MoWO_3_/VO_2_/MoS_2_/Si photodetector and (**b**) responsivity for both MoS_2_/Si and MoWO_3_/VO_2_/MoS_2_/Si photodetectors as a function of the MoS_2_ sputtering time.

Also, the detector responsivity (${R}_{\lambda }$) can be expressed as ${R}_{\lambda }=\Delta I/(A\times P)$, where $\Delta I$ is the difference between the photocurrent and dark current, A is the illuminated area, and P is the UV light power. Figure 13a shows the photocurrent and photocurrent gain of the tested samples under a 365 nm UV illumination source. Figure 13b demonstrates the responsivity in A/W of MoS_2_/Si and MoWO_3_/VO_2_/MoS_2_/Si photodetectors with different MoS_2_ sputtering time (30–240 s). In both photodetectors, the responsivity values reflect nearly linear increases by increasing the sputtering time to 180 s. MoWO_3_/VO_2_/MoS_2_/Si UV detector responsivity shows high values ranging from 0.3 to 4.7 A/W, which corresponds to 30 to 180 s MoS_2_ sputtering time. The optimized value at 180 s is higher than that reported by Li et al. (2.4 mW cm^−2^)^83^. The enhanced photoresponsivity by interfacing the VO_2_ layer may be owing to the film strain include stresses arising from the different thermal expansion coefficients of the VO_2_ and MoS_2_/Si film due to a high deposition/sulphurization temperatures of ~ 400/650 °C and growth stresses arising from crystal structure changes after deposition. Nevertheless, more efficient light absorption involving more *e–h* pairs generation, resulting in higher mobility and more detection capability.

### External quantum efficiency (EQE) and detectivity (${\mathrm{D}}^{*}$)

**Figure 14:**
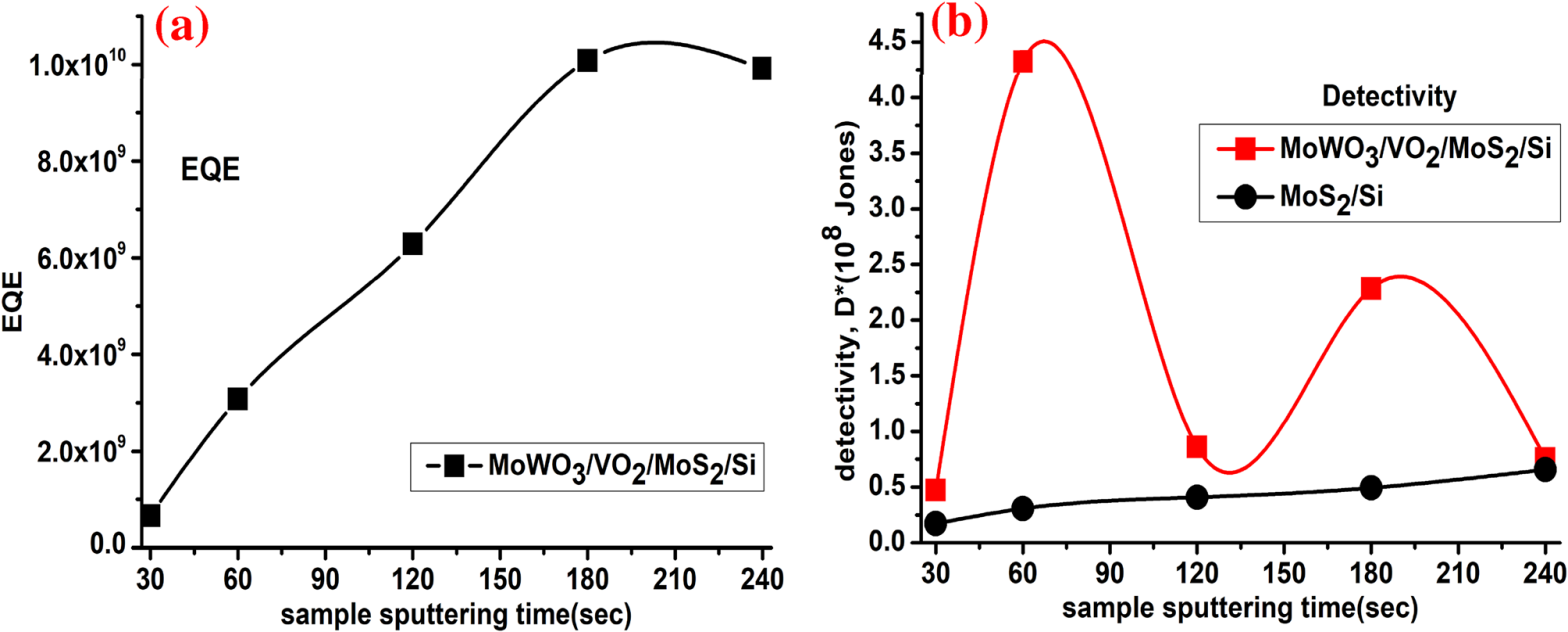
(**a**) The EQE of MoWO_3_/VO_2_/MoS_2_/Si photodetector as a function of the MoS_2_ sputtering time and (**b**) the detectivity of both MoS_2_/Si and MoWO_3_/VO_2_/MoS_2_/Si photodetectors as a function of the MoS_2_ sputtering time.

In order to produce the photocurrent I_ph_, the fraction of the extracted free charge carriers to the photo flux φ_in_ collected at a given energy E_ph_ is called the External Quantum Efficiency, defined by^84^ as $EQE=\frac{hc{R}_{\lambda }}{e\lambda }$, where h is the Planck’s constant (~ 4.135 × 10^−15^ eV s), e is the elementary electron charge (~ 1.602 × 10^−9^ C), c is the light velocity (~ 3 × 10^8 ^m/s), and λ is the excitation wavelength (365 nm). The EQE values as a function of the prepared samples are plotted in Fig. 14a, where EQE varies from 6.6 × 10^8^ to ~ 1.0 × 10^10^ at 365 nm which considered higher than the mesoscopic multilayer MoS_2_ as reported before^85^. Another important figure of merit of a photodetector is the detectable signal^82^, referred by the specific detectivity measured in Jones, which given by ${D}^{*}=\frac{{\left(AB\right)}^{0.5}{R}_{\lambda }}{{i}_{n}} (\mathrm{cm }{\mathrm{Hz}}^\frac{1}{2}{\mathrm{ W}}^{-1})$, where *A* is the effective area of the d in cm^2^, B is the bandwidth, and i_n_ is the measured noise current. If the shot noise from the dark current is the main noise source, the specific detectivity can be simplified as ${D}^{*}=\frac{{R}_{\lambda }{A}^{0.5}}{{(2e{I}_{dark})}^{0.5}}$ ,Where *e* is the charge of an elementary electron^86^. The calculated D* for MoS_2_/Si and MoWO_3_/VO_2_/MoS_2_/Si is depicted in Fig. 14b. D* of MoS_2_/Si device shows a progressive increase with sputtering time from 30 to 240 s. For MoS_2_/Si device, the maximum D* was 0.4 × 10^8^ Jones. While the maximum D* for the MoWO_3_/VO_2_/(60 s)MoS_2_/Si is ~ 4.3 × 10^8^ Jones at RT and applied voltage of 1 V. In contrast the MoWO_3_/VO_2_/MoS_2_/Si with a 30 s deposition time shows D* of 0.47 × 10^8^ Jones.

### Mechanism

Now we turn to the underlying photoresponse mechanism of the VO_2_/MoS_2_/Si as a UV photodetector device. The photoresponse properties of VO_2_/MoS_2_/Si heterojunction can be understood from the energy-band alignment diagram as in Fig. 1b. Due to the free dangling bonds of the surfaces of MoS_2_ film, the MoS_2_/Si heterojunction can be affected by lattice matching. Clearly, the implanting of VO_2_ layer-based UV photodetector was overwhelmingly play an important role in enhancing the Raman signal/intensity, PL intensity, electrical and optoelectronic performance of MoS_2_/Si device. Under the zero-bias condition, an insignificant current was observed due to the high depletion layer at the n–p (MoS_2_-Si) junction which restricts the movement of the carriers. Under VO_2_ interfacing, both positive and negative current increased significantly and the photocurrent I_ph_ of VO_2_/MoS_2_/Si film is much higher than that in MoS_2_-Si, which can be attributed to the more photon absorption on the top of MoWO_3_/VO_2_ layer and larger photocurrent-gain due to higher carrier mobility^87,88^. With increasing the amount of Mo-O (deposition time of 30, 60, 120, 180, and 240 s), the induced current is enhanced due to the more electron–hole pair generation by UV light absorption and the applied voltage shifts dramatically towards negative voltage, which indicates the continuous accumulation of electrons in the vertical VO_2_-MoS_2_ channel as seen from the logarithmic scale current. In the positive voltage region, the MoS_2_/Si n–p structure shows that umpteen electrons are accumulated on the MoS_2_ band which shifts Fermi level near the conduction band. Because 1 V is able to decrease the depletion width and the barrier height, electrons are able to overcome the barrier height through thermionic emission, resulting in a high flux of photocurrent and more efficient photocurrent extraction. It is interesting to observe that under a shorter sputtering time of Mo-O (30, then 60 s), a significant forward photocurrent was observed which did not observe before in MoS_2_/Si structures^52,89^. Meanwhile, with increasing the Mo-O content, MoO_3_ starts to get folded and the reverse photocurrent starts to get highlighted as seen in the semi-logarithmic scale I–V, Fig. 10.

## Conclusion

In summary, the next generation of optoelectronic devices integrates the physics of light-matter interaction of 2D materials at nanoscale for light-harvesting applications and these optoelectronic devices can control the light that converts trions, excitons, and photons to electrical signals. Our approach is based on a high vacuum deposition of Mo-O compound at 400 °C, followed by a sulphurization process in a chemical vapor deposition tube. Here we study the interfacing effect of monoclinic VO_2_ with MoS_2_ film for UV optoelectronic applications. It showed that different thicknesses of the MoS_2_ compound have a direct effect on the Raman, PL, electrical, optoelectronics of MoS_2_ peaks. A redshift was observed in Raman spectra with a high electronic coupling between VO_2_ and MoS_2_ for the case of 180 s sputtering time. Photoluminescence measurements showed that the intensity of the trion peak has a higher intensity than the A-exciton peak for MoS_2_/Si structure. On the other hand, the opposite case was observed for the VO_2_/MoS_2_/Si device. Current–voltage, response/recovery time, external quantum efficiency, time-resolved photocurrent, and detectivity, photocurrent gain, photo-responsivity of VO_2_/MoS_2_/Si device have been demonstrated. It’s shown that the increase of the deposition time of MoS_2_ from 30 to 240 s enhances the photo-absorption, photo-responsivity, and external quantum efficiency of the MoWO_3_/VO_2_/MoS_2_/Si device due to the associated folding effects of MoO_3_. These results show a multiplexed photodetector fabrication technique of high reproducible and scalable process based on CVD and PVD system and draw high attention towards the interfacing effects of strongly correlated oxide films MoS_2_ devices.

## Materials and methods

### Device fabrication

Preparation of MoS_2_ layer on p-type Si substrate has prepared through two steps in physical vapor deposition (PVD)—Radio Frequency magnetron sputtering system, followed by chemical vapor deposition (CDV) process. Si substrates were cleaned through many steps; firstly, kept in NH_4_OH-H_2_O_2_ solution diluted with de-ionized (DI) water for 5 min at 75 °C, then rinsed with DI water for 5 min. After that, they left in HF (%5) solution for 5 s, then rinsed in DI water and dried with high purity N_2_. Immediately, the cleaned Si-substrates transferred to a 3 × 10^−7^ Torr RF magnetron sputtering system (VAKSIS Midas 3M1T). In-situ Ar-plasma source has activated for 10 min at a power of 100 W and low pressure of 6 × 10^−3^ Torr at room temperature to activate the Si surface. Mo-O thin films were grown using a 3-inch. pure molybdenum target (99.9%) utilizing Ar plasma as a carrier gas and O_2_ as a reactive gas. The substrate temperature was stabilized at 400 °C for more than 30 min before the deposition process with steps of 100 °C/30 min. The O_2_ and Ar flow rates were kept constant, whereas O_2_/(Ar + O_2_) of ¼ ratio. The deposition was carried out at 5 × 10^−3^ Torr and 137 W with different sputtering times of 30, 60, 120, 180, and 240 s. The system was kept to cool down normal up to the room temperature (RT), then immediately transferred to the two-zone CVD quartz chamber (MTI-OTF 1200 system) for the sulphurization process. The as-deposited molybdenum oxide (Mo-O) thin films transferred to the center of the CVD furnace and the temperature is raised to 650 °C. Sulphur powder (0.5 g) is put in a ceramic boot with 100sccm high purity Ar source. An external heating belt with a distance of 50 cm to the substrate was used to evaporate the sulphur for 22 min. Then, the system cooled down until RT with the same flow rate of Ar (100 sccm).

After forming the MoS_2_ layer, a thin layer of monoclinic VO_2_ has grown. The same sputtering system was used with a 190 W deposition power and Ar/O_2_ ratio was 41/2.2 sccm, while the deposition time was set to produce 50 nm film thickness. Then the samples were in-situ annealed at 400 °C for 2 h with 50 sccm Ar flow. A protective and anti-reflection thin layer of Mo_0.2_W_0.8_O_3_ was deposited on the surface of VO_2_ as optimized in our previous work^34^. High vacuum thermal evaporation system was used to deposit aluminum and gold–palladium that used as a back and front contacts, respectively.

### Device characterization

The crystal structures were analyzed using Grazing Incidence X-ray diffraction (XRD GNR ADP PRO 2000), with CuKα (λ = 1.5405 Å) radiation source with a step of 0.01. VO_2_ layer was deposited at a high vacuum condition to ensure its high crystallinity and low semiconductor-metallic phase transition. Parameters such as space group, diffraction peaks, angles, Wyckoff position of vanadium (V) and oxygen (O) atoms, ratio of O:V, and oxygen vacancy concentrations were calculated from refinement analysis. The refinement calculations were done using Match and Fullprof Suite program. Moreover, the refined structures were plotted in a three-dimensional view using 3D visualization VESTA program. The surface morphology was recorded using scanning electron microscopy (SEM) TESCANMAIA3 XMU. Atomic Force Microscopy (AFM) has been used to investigate the surface topography, roughness, and grain mapping. Each sample was characterized by XE-6 AFM (Park Systems Corp., Suwon-Korea) that controlled with XEP software for data acquisition and XEI software for image analysis and processing. AFM images were obtained through a 0.5 × 0.5 μm area (x–y accessible area) at a 0.5 Hz scan rate. Measurements were taken with a non-contact mode using a PPP-NCHR silicon cantilever consisting of tip radius < 10 nm and 42 N/m force constant (Nanosensors TM, Neuchâtel-Switzerland). Raman measurements and photoluminescence (PL) spectra were carried out using Renishaw in Via Confocal Raman microscope with a 532 nm laser beam, while an incident laser power of 3 mW was chosen to acquire a single Raman spectrum. The temperature-resistance measurement of monoclinic and high crystalline nanostructure VO_2_ thin film has been performed using a four-probe measurement system connected to a heating stage ranging from RT to 100 °C. The electrical and optoelectronics measurements were measured using 2450 Kethley Source -Meter and 365 nm ultraviolet (UV) light lamp for optoelectronic measurements.

## Supplementary information

Supplementary Information.
