# Supplementary material for: Efficient MoWO3/VO2/MoS2/Si UV Schottky photodetectors; MoS2 optimization and monoclinic VO2 surface modifications
Source: Sci Rep. 2020 Sep 28;10:15926. doi: 10.1038/s41598-020-72990-9 (PMC7522211; doi:10.1038/s41598-020-72990-9)
Supplement: Supplementary file 1 — Supplementary Information. [file 41598_2020_72990_MOESM1_ESM.docx]

Supplementary data

Efficient MoWO_3_/VO_2_/MoS_2_/Si UV Schottky photodetectors; MoS_2_ optimization and monoclinic VO_2_ surface modifications

Mohamed A. Basyooni^1, 4^, Shrouk E. Zaki^1^, Mohamed Shaban^2*^, Yasin Ramazan Eker^3, 4^ and Mucahit Yilmaz ^1^

^1^Nanophysics Laboratory, Department of NanoScience and NanoEngineering, Institute of Science and Technology, University of Necmettin Erbakan, Konya 42060, Turkey

^2^Nanophotonics and Applications Laboratory, Department of Physics, Faculty of Science, Beni-Suef University, Beni-Suef 62514, Egypt

^3^Department of Metallurgy and Material Engineering, Faculty of Engineering and Architecture, Necmettin Erbakan University, Konya 42060, Turkey

^4^Science and Technology Research and Application Center (BITAM), University of Necmettin Erbakan, Konya, 42060, Turkey

* Corresponding author: Mohamed Shaban

E-mails: mssfadel@aucegypt.edu; [mohamed.fadel@science.bsu.edu.eg](mailto:mohamed.fadel@science.bsu.edu.eg)

**SEM characterization**

The scanning electron microscopy (SEM) was used to investigate the surface morphology of the prepared VO_2_, MoO_3,_ and Mo_0.2_W_0.8_O_3_ thin films as shown in Figure S1. Figure S1 (a), (b), and (c) show the surface morphology of VO_2_, MoO_3_, and Mo_0.2_W_0.8_O_3_ thin films, respectively. The high deposition vacuum and relatively high sputtering temperature provide us with nanostructured and uniform thin films as seen in Figure S1 (a). The optimized 400^o^C deposition temperature of Mo-O thin film resulting in scattered nanosheets as in Figure S1 (b). This film used to form MoS_2_ films through a chemically sulphurised process in the chemical vapor deposition (CVD) system. While Figure S1 (c) shows a small grain in Mo_0.2_W_0.8_O_3_ thin film. Figure S2 shows the SEM (a, b, c, d, and e) of the prepared MoS_2_- Si thin films with a sputtering time of 30, 60, 120, 180, and 240 sec, respectively. In Figure S2 (a), a small, ordered and homogenous nano-sheets started to grow, meanwhile, in Figure S2 (b), the scattered-nanosheets start to grow with small agglomerations. While larger nanoparticles were shown in Figure S2 (d) and (e) corresponding to 180 and 240sec sputtering time. Samples with loge sputtering time show an agglomeration of nanoparticles with different sizes less than 100nm. It seems that with increasing the deposition time of MoO_3_, the accumulated nanoparticles show bigger sizes, consequently higher roughness factors. Large scale MoS_2_ thin films have been studied in our previous work by combining CVD and sputtering techniques [1]. SEM images (Fig. S2, supplementary data) are provided to illustrate a homogenous and approximately uniform nanoparticles distribution. The SEM images in Fig. S2 (supplementary data) are provided to illustrate the homogeneity and approximate uniform surface distribution of nanoparticles, particularly for sputtering time < 180 sec.


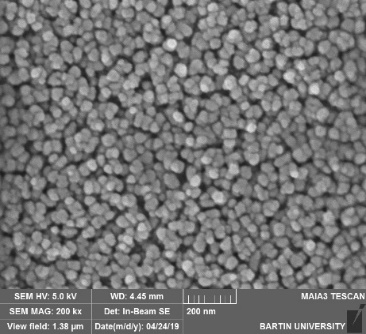


**(a)**


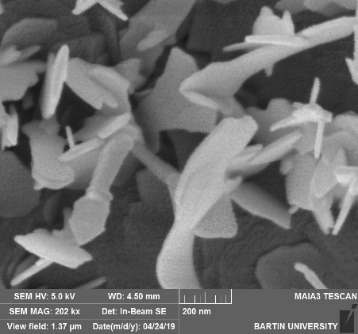


**(b)**


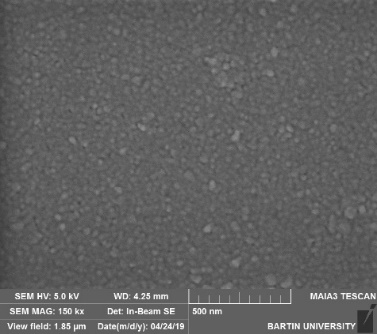


**(c)**

Fig. S1. SEM images of each deposited layer; surface morphology of (a) VO_2_, (b) MoO_3,_ and (c) Mo_0.2_W_0.8_O_3_ thin films.


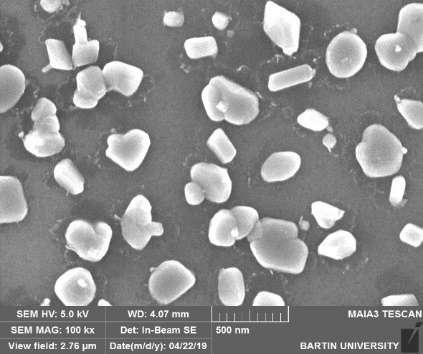


**(d)**


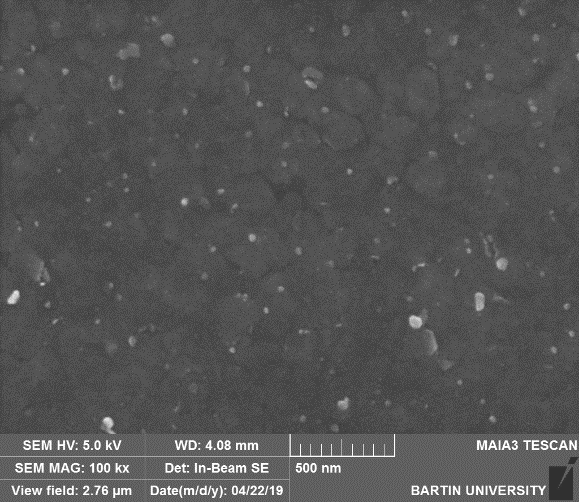


**(a)**


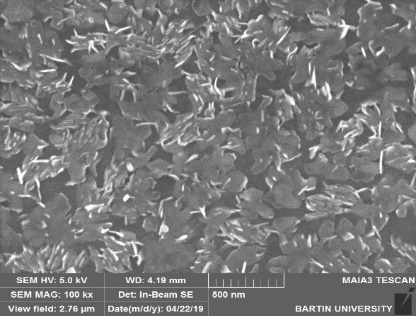


**(b)**


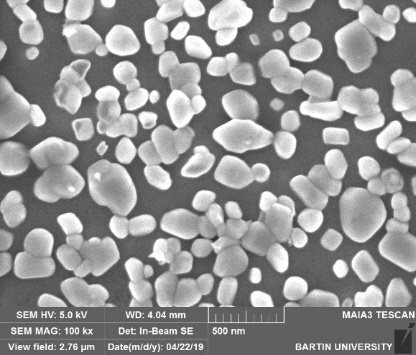


**(e)**


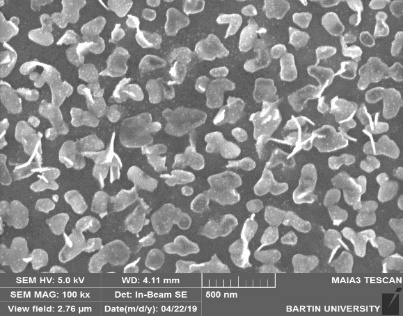


**(c)**

Fig. S2. SEM images of the prepared MoS_2_ thin films at different sputtering times; (a) 30, (b) 60, (c) 120, (d) 180, and (e) 240 sec.


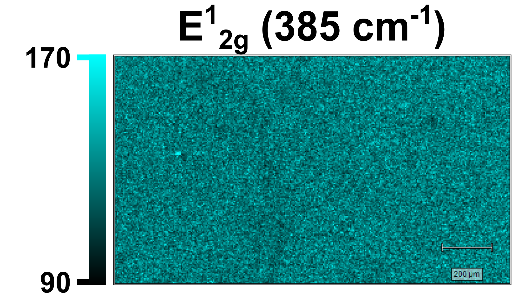

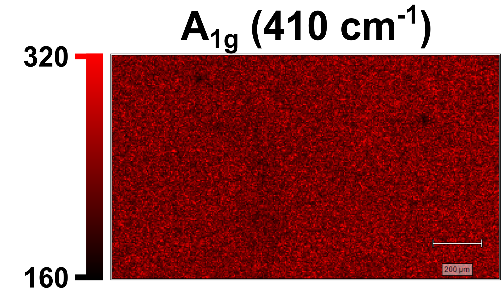

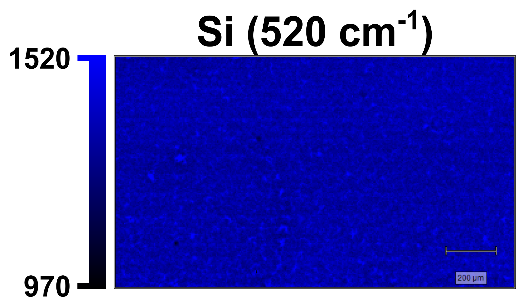


**(a)**


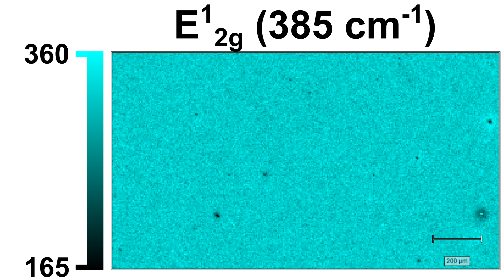

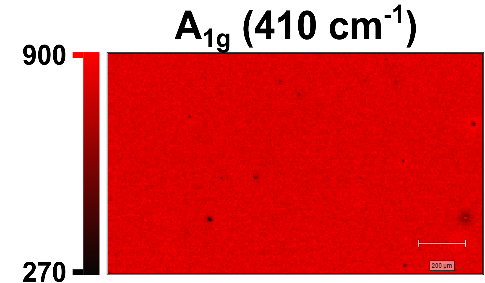

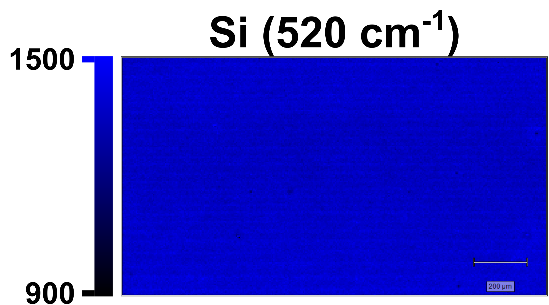


**(b)**


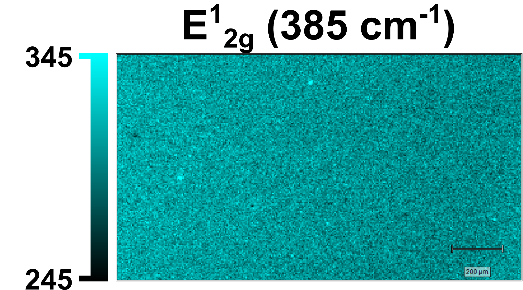

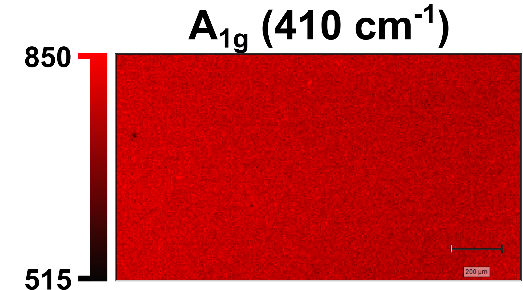

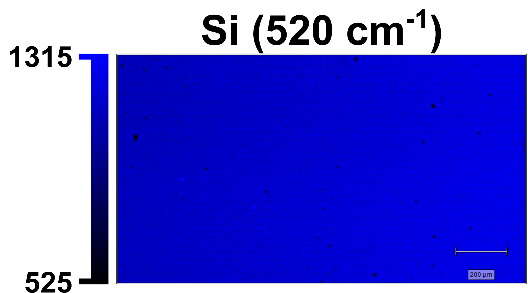


**(c)**


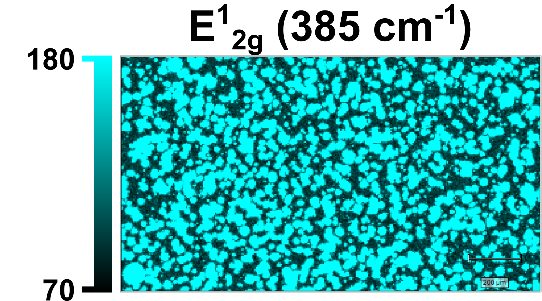

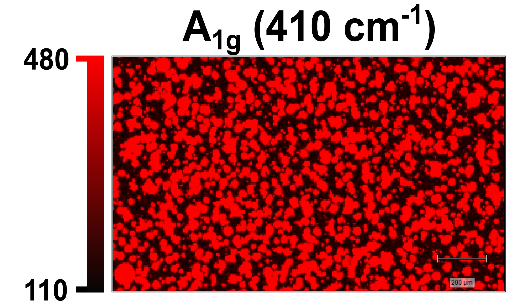

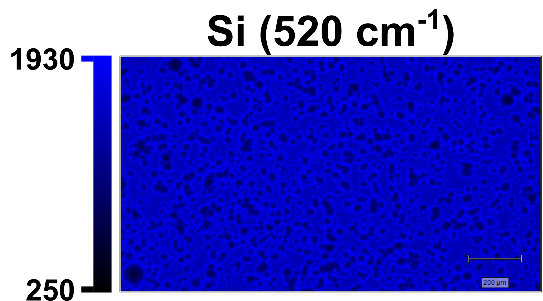


**(d)**


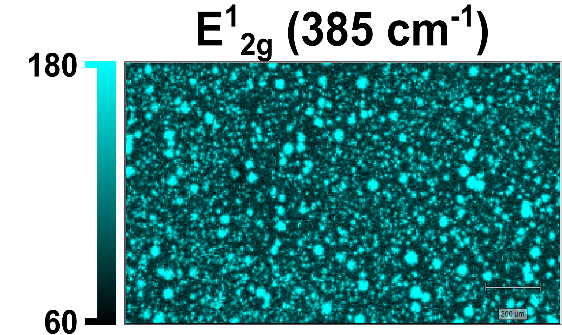

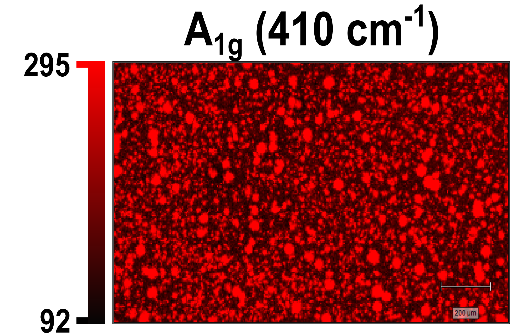

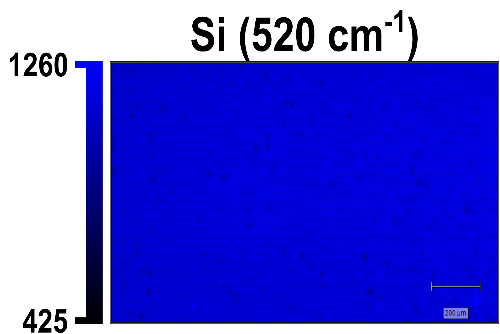


**(e)**

Fig. S3 (a, b, c, d, and e) shows the Raman mapping of MoS_2_ at different sputtering times of Mo-O of 30, 60, 120, 180, and 240sec, respectively. [ This figure is presented by OriginPro8.6; ;   <https://www.originlab.com/index.aspx?go=Company/NewsAndEvents/PressRoom&pid=1820>]

For more details about intensity distribution versus position, Raman mappings of $E_{2g}^{1}, A_{1g}$ and Si peaks of MoS_2_/Si at different sputtering times of Mo-O (30, 60, 120, 180, and 240 sec) are investigated at 385, 410, and 520 cm^-1^, respectively, and shown in Fig. S3 (a, b, c, d, and e). The Raman mapping was realized at a large area (1.6mm x 1mm) with 320x200 data points using the Leica microscope at 5X magnification to provide evidence about the ability to scale up the MoS_2_ thin films. The wavelength, power, and integration time of the used laser were 532 nm, 3 mW, and 1 sec, respectively. This analysis illustrated that 30, 60, and 120 sec samples show high homogeneity over the full scale (1.6x1 mm^2^). Although 180 sec sample shows regular agglomerations of nanoparticles for both E_2g_ and A_1g_ positions, the 240 sec sample shows irregular clusters. The scaling up of MoS_2_ can, therefore, be demonstrated until sputtering time < 180 min. Similar reports have grown MoS_2_ using magnetron sputtering through one or two steps such as Shijie Wang et al. [2], Rongsheng Chen et al. [3], K.-W. Ang et al. [4], Wan-Gyu Lee et al. [5], Jianrong Xiao et al. [6], Craig E. Banks et al.[7], and Peiling Ke et al.[8].


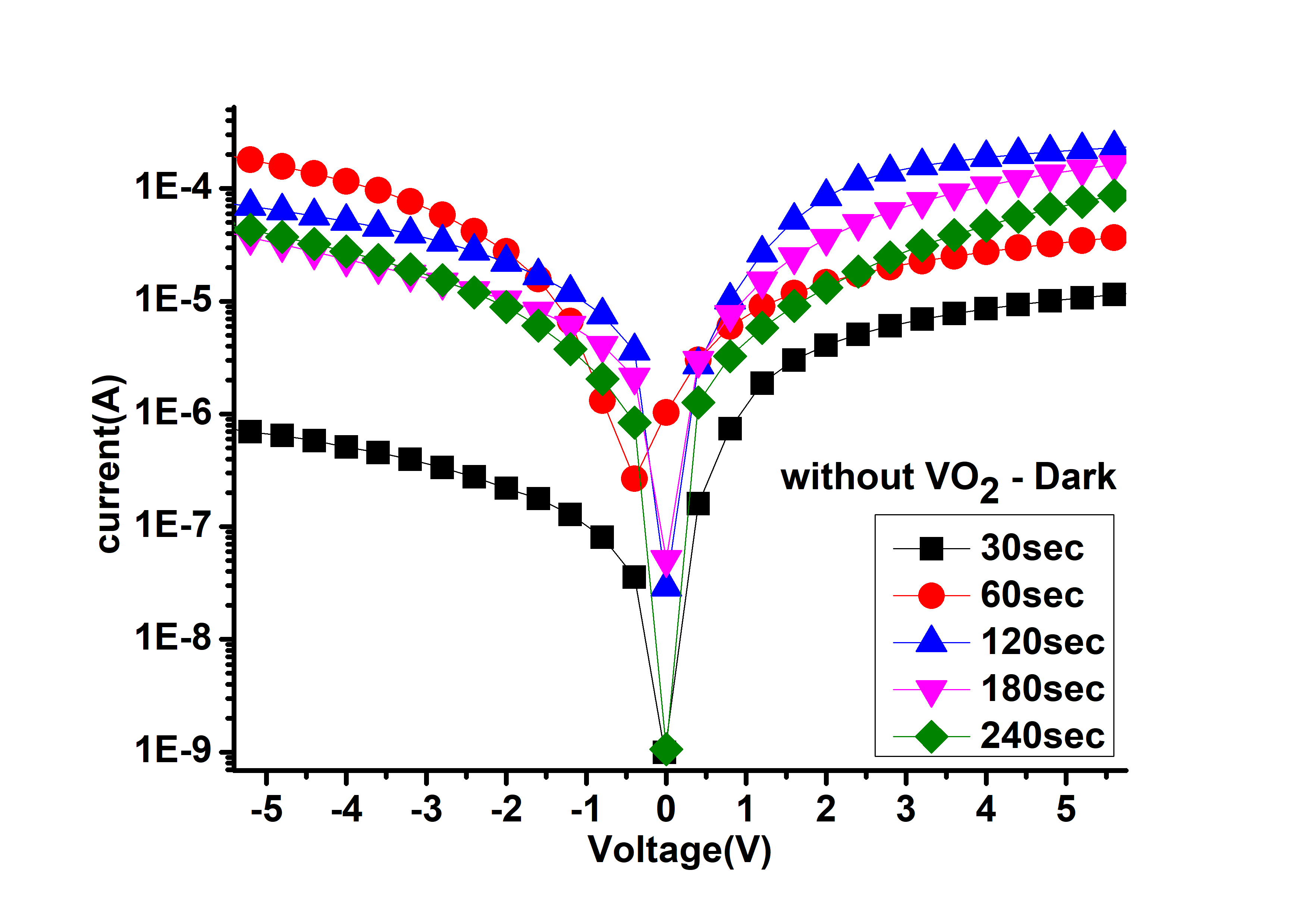


**(b)**


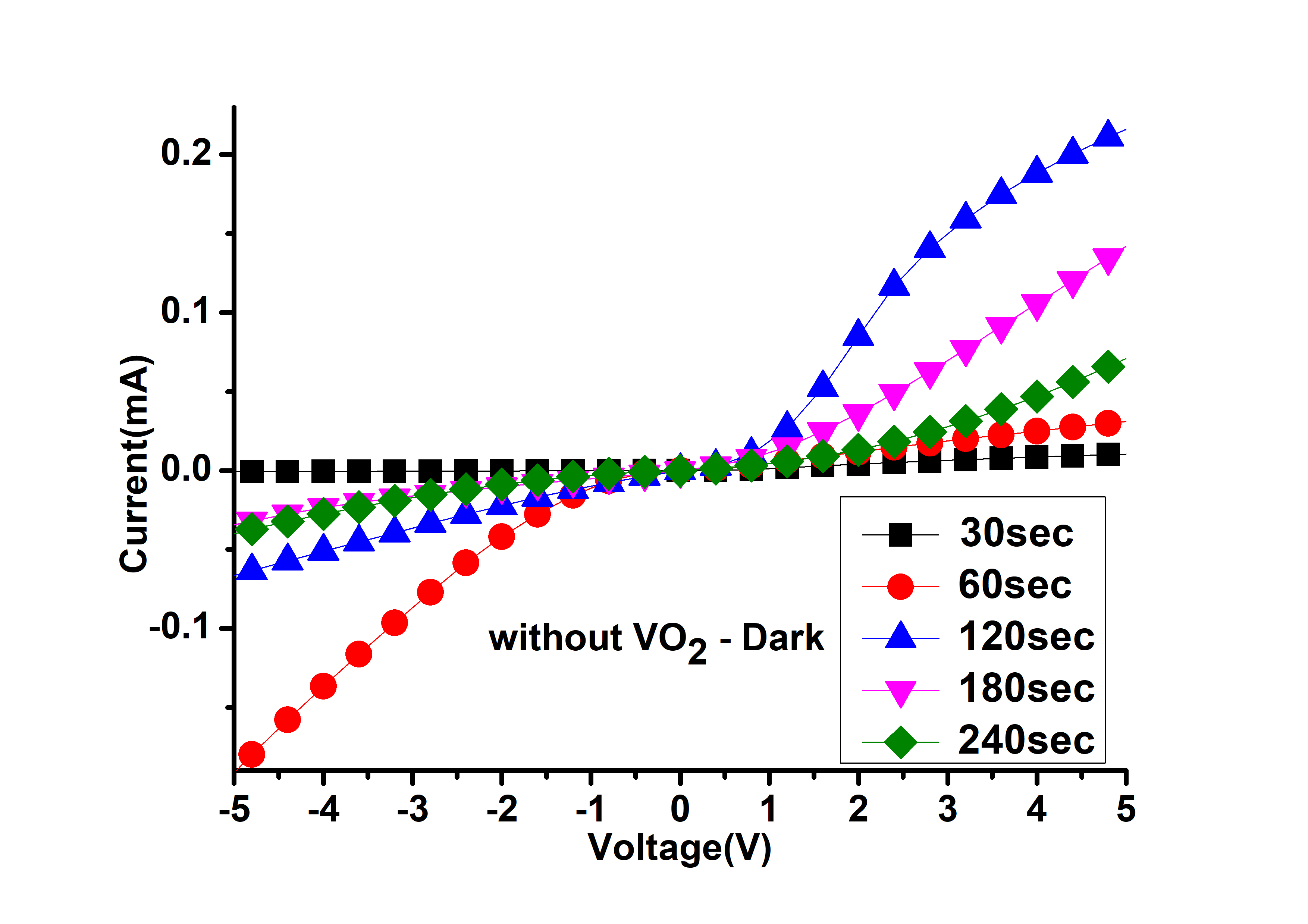


**(a)**

Fig. S4 (a) the linear and (b) semi-logarithmic scale current-voltage characteristics of MoS_2_/Si device under the dark condition with different sputtering times of MoS_2_ layer; 30, 60, 120, 180, and 240 sec.

Figure S4 shows the linear and semi-logarithmic scale current-voltage characteristics of MoS_2_/Si device without VO_2_ layer under the dark condition with different sputtering times of MoS_2_ layer; 30, 60, 120, 180, and 240sec. The positive part shows an increase in the associated dark current with increasing the sputtering time from 30 to 60 sec. While at 120 sec, a jump in the forward dark current is observed due to the related folding-effects in MoS_2_. Folding effect decreases the interlayer coupling and enhances the photoluminescence emission yield of A- and B- exciton peaks as seen in Figure 5 [9,10]. Whereas instability measurements were observed at the negative bias part. Similar behavior was previously reported for the MoS_2_ field-effect transistor (FET) by K. Cho *et al.,* [11,12]. The large increase in the negative dark current for MoS_2_(60 sec) may be attributed to the release of charges that were trapped on MoS2 's surface at the interface trap sites (oxygen sites). The highest reverse dark current, which suggests the lowest potential barrier, was observed at 60 sec. This may be ascribed to the values of the optical band gaps as shown in our previous study, whereas the 60 sec MoS_2_-Si thin film displayed optical band gaps of 1.75 and 2.01 eV [13]. By increasing the sputtering time the reverse dark current decreases and almost becomes identical for sputtering time ≥ 180 sec, as shown in Fig.S4(a,b).


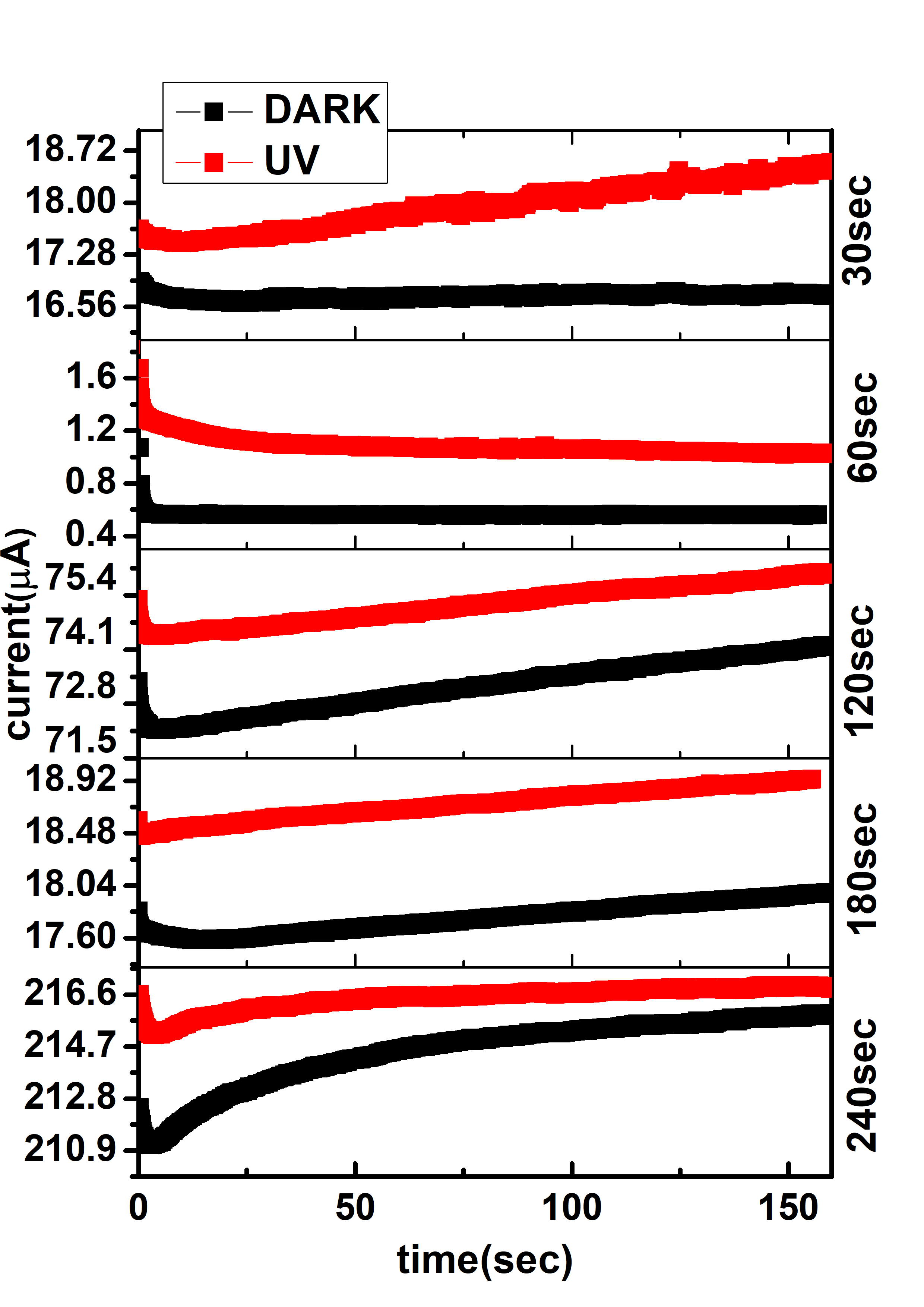


Fig. S5. The photocurrent-time (I-t) characteristics under dark and UV illumination of Mo_0.2_W_0.8_O_3_/VO_2_/MoS_2_-Si photodetectors with different MoS_2_ sputtering times (30, 60, 120, 180, and 240 sec).

Figure S5 shows the dynamic behavior of current with time (I-t)@1V under dark and UV illumination of the designed Mo_0.2_W_0.8_O_3_/VO_2_/MoS_2_-Si heterostructure devices at different Mo-O sputtering time of 30, 60, 120, 180, and 240 sec, respectively. All samples show symmetric kinetics through 150 sec with remarkable differences in the generated photocurrents. The engineered MoWO_3_/VO_2_/(60sec)MoS_2_/Si heterostructure showed the lowest photocurrent, the highest resistance, and the most stable curve overtime under dark and UV illumination, referring to the dependence of VO_2_ surface coupling and strain-induced optical modulation on the sputtering time of the Van der Waals MoS_2_/Si heterostructure.

**References**

[1] J.D. Hwang, W.T. Chang, Y.H. Chen, C.Y. Kung, C.H. Hu, P.S. Chen, Suppressing the dark current of metal-semiconductor-metal SiGe/Si heterojunction photodetector by using asymmetric structure, Thin Solid Films. 515 (2007) 3837–3839. DOI:10.1016/j.tsf.2006.10.017.

[2] J. Tao, J. Chai, X. Lu, L.M. Wong, T.I. Wong, J. Pan, Q. Xiong, D. Chi, S. Wang, Growth of wafer-scale MoS2 monolayer by magnetron sputtering, Nanoscale. 7 (2015) 2497–2503. DOI:10.1039/c4nr06411a.

[3] W. Zhong, S. Deng, K. Wang, G. Li, G. Li, R. Chen, H.S. Kwok, Feasible route for a large area few-layer MoS2 with magnetron sputtering, Nanomaterials. 8 (2018). DOI:10.3390/nano8080590.

[4] Z.P. Ling, R. Yang, J.W. Chai, S.J. Wang, W.S. Leong, Y. Tong, D. Lei, Q. Zhou, X. Gong, D.Z. Chi, K.-W. Ang, Large-scale two-dimensional MoS_2 photodetectors by magnetron sputtering, Opt. Express. 23 (2015) 13580. DOI:10.1364/oe.23.013580.

[5] S. Hussain, J. Singh, D. Vikraman, A.K. Singh, M.Z. Iqbal, M.F. Khan, P. Kumar, D.C. Choi, W. Song, K.S. An, J. Eom, W.G. Lee, J. Jung, Large-area, continuous and high electrical performances of bilayer to few layers MoS2 fabricated by RF sputtering via post-deposition annealing method, Sci. Rep. 6 (2016). DOI:10.1038/srep30791.

[6] M. Qi, J. Xiao, C. Gong, Thermal annealing effects on the electrophysical characteristics of sputtered MoS2 thin films by Hall effect measurements, Semicond. Sci. Technol. 34 (2019). DOI:10.1088/1361-6641/ab09a5.

[7] S.J. Rowley-Neale, M. Ratova, L.T.N. Fugita, G.C. Smith, A. Gaffar, J. Kulczyk-Malecka, P.J. Kelly, C.E. Banks, Magnetron Sputter-Coated Nanoparticle MoS2 Supported on Nanocarbon: A Highly Efficient Electrocatalyst toward the Hydrogen Evolution Reaction, ACS Omega. 3 (2018) 7235–7242. DOI:10.1021/acsomega.8b00258.

[8] L. Gu, P. Ke, Y. Zou, X. Li, A. Wang, Amorphous self-lubricant MoS 2 -C sputtered coating with high hardness, Appl. Surf. Sci. 331 (2015) 66–71. DOI:10.1016/j.apsusc.2015.01.057.

[9] A. Castellanos-Gomez, H.S.J. van der Zant, G.A. Steele, Folded MoS2 layers with reduced interlayer coupling, Nano Res. 7 (2014) 1–7. DOI:10.1007/s12274-014-0425-z.

[10] T. Jiang, H. Liu, D. Huang, S. Zhang, Y. Li, X. Gong, Y.R. Shen, W.T. Liu, S. Wu, Valley and band structure engineering of folded MoS 2 bilayers, Nat. Nanotechnol. 9 (2014) 825–829. DOI:10.1038/nnano.2014.176.

[11] K. Cho, W. Park, J. Park, H. Jeong, J. Jang, T.Y. Kim, W.K. Hong, S. Hong, T. Lee, Electric stress-induced threshold voltage instability of multilayer MoS2 field effect transistors, ACS Nano. 7 (2013) 7751–7758. DOI:10.1021/nn402348r.

[12] K. Cho, T.Y. Kim, W. Park, J. Park, D. Kim, J. Jang, H. Jeong, S. Hong, T. Lee, Gate-bias stress-dependent photoconductive characteristics of multi-layer MoS2 field-effect transistors, Nanotechnology. 25 (2014) 155201–155208. DOI:10.1088/0957-4484/25/15/155201.

[13] A. Karataş, M. Yılmaz, Molybdenum disulfide thin films fabrication from multi-phase molybdenum oxide using magnetron sputtering and CVD systems together, Superlattices Microstruct. 143 (2020). DOI:10.1016/j.spmi.2020.106555.
